# LLM and CQL computable phenotypes for myocarditis adverse event feature and outcome determinations using patient FHIR data: a feasibility study

**DOI:** 10.3389/fdgth.2026.1903755

**Published:** 2026-09-08

**Authors:** Fraser W. Gaspar, Seth B. Roberts, Francis X. Campion, Lynda Hoeksema, Jessica Kasirsky, Raymond Duncan, Matthew Sonesen, Renier Estiandan, Khyzer B. Aziz, Julie A. Riddler, Jessica Zhou, Hussein Ezzeldin

**Affiliations:** 1 Center for Transforming Health, MITRE Corp., McLean, VA, United States; 2 Departments of Enterprise Information Services and Pediatrics, Cedars-Sinai Health System, Los Angeles, CA, United States; 3 Johns Hopkins Medicine, Baltimore, MD, United States; 4 Center for Biologics Evaluation and Research, Food and Drug Administration, Silver Spring, MD, United States

**Keywords:** adverse event, CQL, FHIR, LLM, surveillance, pharmacovigilence, health information network

## Abstract

**Background:**

Post-market surveillance for adverse events of special interest is often constrained by key diagnostic elements being captured primarily in unstructured clinical notes and reports. This feasibility study evaluated large language model (LLM) and clinical quality language (CQL) methodologies to perform phenotype feature extraction and outcome determination on Fast Healthcare Interoperability Resources (FHIR) data.

**Methods:**

Two health systems identified patients with an mRNA COVID-19 vaccination and an inpatient myocarditis diagnosis within 42 days. Patient FHIR data was retrieved via a Health Information Exchange and converted into narrative text (“textified”). Llama3.3 70B Instruct answered yes/no Brighton Collaborative–based myocarditis feature questions; answers were written back as FHIR Observation resources to enhance each patient bundle. Three methods were compared: simple CQL (structured data only), LLM (textified FHIR data), and enhanced CQL (CQL on enhanced bundles). Three clinicians provided gold-standard determinations. An optimization set (*n* = 10) supported pipeline refinement; performance was evaluated in a test set (*n* = 25).

**Results:**

In the test set, simple CQL performed poorly for identifying cardiac and non-specific symptoms [Matthews Correlation Coefficient (MCC): 0.08; F1: 0.02] but excellently for cardiac biomarkers (MCC:0.83; F1: 0.88). The LLM achieved excellent performance identifying symptoms and imaging features (e.g., echocardiogram abnormalities MCC: 0.86) but weaker performance for biomarkers (MCC: 0.54). Enhanced CQL had excellent feature identification performance across most domains. Both LLM and enhanced CQL correctly determined the myocarditis outcome for 23 of 25 patients.

**Conclusions:**

In this small feasibility study, structured and unstructured FHIR data provided the computable phenotypes using an LLM with sufficient clinical information for myocarditis feature identification and outcome determination.

## Introduction

The identification, validation, and reporting of adverse events is a time and labor-intensive process. The U.S. Food and Drug Administration (FDA) conducts post-market safety surveillance for vaccines and biologics through programs that use real-world clinical data. In 2017, the FDA's Center for Biologics Evaluation and Research (CBER) started the Biologics Effectiveness and Safety Innovative Methods (BEST IM) exchange program to advance the agency's post-market surveillance by leveraging Fast Healthcare Interoperability Resources (FHIR)-based exchanges via health information networks (HIN) (1). BEST IM initiated novel studies utilizing a HIN and FHIR data (2). Initial BEST IM studies utilized Clinical Quality Language (CQL) to specify computable phenotypes on structured data; however, performing adverse event detection only utilizing structured data did not show robust validation results (3, 4). For many phenotypes, critical diagnostic elements—including the characterization of symptoms, imaging narratives, and biopsy findings—are frequently documented in clinical notes or diagnostic reports rather than fully captured in diagnosis, observation, or procedure codes.

Post-market vaccine safety surveillance is a global challenge because clinically important adverse events are often identified from routine care data that were not collected primarily for research or regulatory review. Although structured elements such as diagnosis codes and laboratory results can support scalable analyses, key diagnostic details are frequently documented only in free-text clinical notes, imaging reports, and discharge summaries. This problem is not unique to the United States and affects surveillance efforts across healthcare systems with varying levels of digital maturity.

Given the limitations of structured-data–only approaches previously demonstrated (3), leveraging large language models (LLMs) to extract features from unstructured FHIR data may substantially improve pharmacovigilance practices and improve patient safety (5). The widespread availability of large language models has improved the ability of systems to extract relevant clinical information from unstructured data (6–9). Despite this rapid progress, the integration of LLM-derived features into standards-based, FHIR-driven pipelines for post-market safety surveillance has not been systematically evaluated.

In this study, we compared three approaches to identifying phenotype adverse-event features and outcome using messenger ribonucleic acid (mRNA) COVID-19 vaccination from FHIR data as a use case: 1) a general-purpose LLM operating over “textified” patient FHIR bundles, 2) CQL queries over structured FHIR data, and 3) a hybrid method that writes LLM-extracted features back into the patient's FHIR bundle to enable enhanced CQL-based identification. We use post-vaccination myocarditis as an illustrative phenotype because its determination often depends on integrating symptoms, biomarkers, electrocardiographic findings, and imaging interpretations, many of which are incompletely represented in coded data alone. The objective of this work is to evaluate methods for feature extraction and case determination against a standardized case definition, not to infer causality between vaccination and myocarditis.

## Materials and methods

### Data

Two health systems provided the FDA FHIR data to investigate our three approaches. Given that the goal of this pilot was to validate methods of identifying adverse event features (data elements) and myocarditis, the study protocol requested health systems to send initial lists of “patients of interest”, i.e., patients who may have experienced myocarditis after COVID-19 mRNA vaccination. To find the patients of interest, exposure and outcome value sets were sent to health systems, and the health systems queried their Epic Clarity databases to identify potential patients. The health systems sent back to the FDA a list of all patients who were at least 12 years old: 1) had received a COVID-19 mRNA vaccination between December 2020 to May 2025 and 2) were diagnosed with myocarditis between December 2020 to July 2025.

Preliminary inclusion/exclusion logic was applied to this initial cohort to filter down to patients whose: 1) a first myocarditis diagnosis did not imply an etiology and occurred within 42 days after a COVID-19 mRNA vaccine administration and; 2) the myocarditis diagnosis occurred during an inpatient visit.

Using the medical record number of potential myocarditis patients, patient FHIR data was requested via a Clinical Data Exchange (CDex) Task Data Request (10) to eHealth Exchange (eHx) for each patient. The Task Data Request specified that the health systems should send back the following resources, within the limited case window from start to end dates provided in the request: AllergyIntolerance, Condition, DiagnosticReport, DocumentReference, Encounter, Immunization, Location, Medication, MedicationRequest, Observation, Patient, Practitioner, Procedure. eHx processed the Task Data Request and queried for the requested patient resources at the appropriate health system. eHx temporarily stored the FHIR resources until the FDA pulled the FHIR resources and loaded the patient’s data into a secure Postgres database, accessible via a HAPI FHIR server. Once in the HAPI FHIR server, health system provided vocabularies and units were confirmed to match value set and logic expectations.

### Myocarditis adverse event definition

The clinical features and outcome determination algorithms were derived from Safety Platform for Emergency Vaccines (SPEAC) project's interpretation of Brighton Collaborative's 2022 myocarditis case definition of an adverse event after vaccination (11, 12). The SPEAC interpretation has a series of yes/no questions that are algorithmically combined to produce a myocarditis outcome determination. The questions cover cardiac and non-specific symptom presence, elevated cardiac biomarkers, imaging results, and biopsy findings. Elevated troponin T, troponin I, and creatine kinase-myocardial band (CK-MB) were defined as being above 0.01 ng/mL, 0.015 ng/mL, and 5 ng/mL, respectively (13, 14). Outcome determinations were categorized as either ‘definite’ or ‘probable’ when they aligned, respectively, to the Brighton Collaborative level 1 or 2 case definitions. All other outcome determinations were categorized as ‘not present.

### LLM pipeline

The LLM used in this part of the work was an instance of Llama3.3 70B Instruct, hosted on a secure server in the FDA High-performance Integrated Virtual Environment. FHIR resources were converted to narrative paragraphs by selecting a subset of the most important attributes, in order to help the LLM focus on the most critical information. The process of translating FHIR data to narrative text is referred to as “textification” in this study. Textification is an entirely algorithmic process whereby essential clinical information are extracted from the FHIR patient bundle; these values are then inserted into a string template to create narrative paragraphs holding the essential information contained in each FHIR resource. Any missing attributes are given a value of “unknown”. No LLMs were used for textification. All the resources in a filtered patient bundle were textified and put together into a single Word document that clinicians could review. The same textified document was provided to the LLM to reason over when answering the key questions.

Even after filtering the patient bundles for relevant resources, the textified patient data was often too large to fit in the context window for the LLM. To address this issue, the textified data for a single patient was split into multiple “cuts” or chunks, each with ∼20,000 or fewer tokens. The cuts were created so that textified FHIR resources were always contained within a single cut of data, never split across two or more cuts. In very rare cases, certain DocumentReference or DiagnosticReport resources, when textified, contained many more than 20,000 tokens, e.g., 40,000 or 60,000 tokens. Investigation revealed these resources were aggregated documents that duplicated information available elsewhere in the patient data (e.g., a discharge summary appended to a letter addressed to a referring physician). Thus, in these rare cases, the extremely large resources were excluded from the textified data to prevent resource sections from being analyzed in two chunks, potentially losing valuable context.

The pipeline then prompted the LLM to answer each of the key clinical questions, using each cut of patient data. Each key question was framed such that the LLM determined whether the corresponding clinical finding was present, the estimated date of occurrence, whether the finding occurred within a specified diagnostic window, and the rationale for the LLM response. The questions were accompanied by instructions to ensure the LLM provided responses in a structured JSON format, avoiding hallucination and relying solely on the provided patient data. The instructions were also designed to encourage “chain of thought” reasoning, where a step-by-step reasoning process is explicitly articulated to connect the problem to its final answer. Prompts for each feature are included in the Supplemental Material.

The Llama LLM instance was served locally using vLLM and was initialized with a context window of 65,000 tokens, BitsAndBytes quantization, a random seed of 42, and tensor parallelism across 4 GPUs. Sampling parameters were not explicitly set per request; the model's generation_config.json defaults of temperature=0.6 and top-*p* = 0.9 were applied by the server. No maximum output token limit was specified per request; output length was bounded by the context window minus the input prompt length. LLM responses to individual questions were aggregated across chunks as follows: any ‘yes’ response across chunks was taken as the aggregate answer to the question, otherwise the aggregate answer was ‘no’.

**Figure 1:**
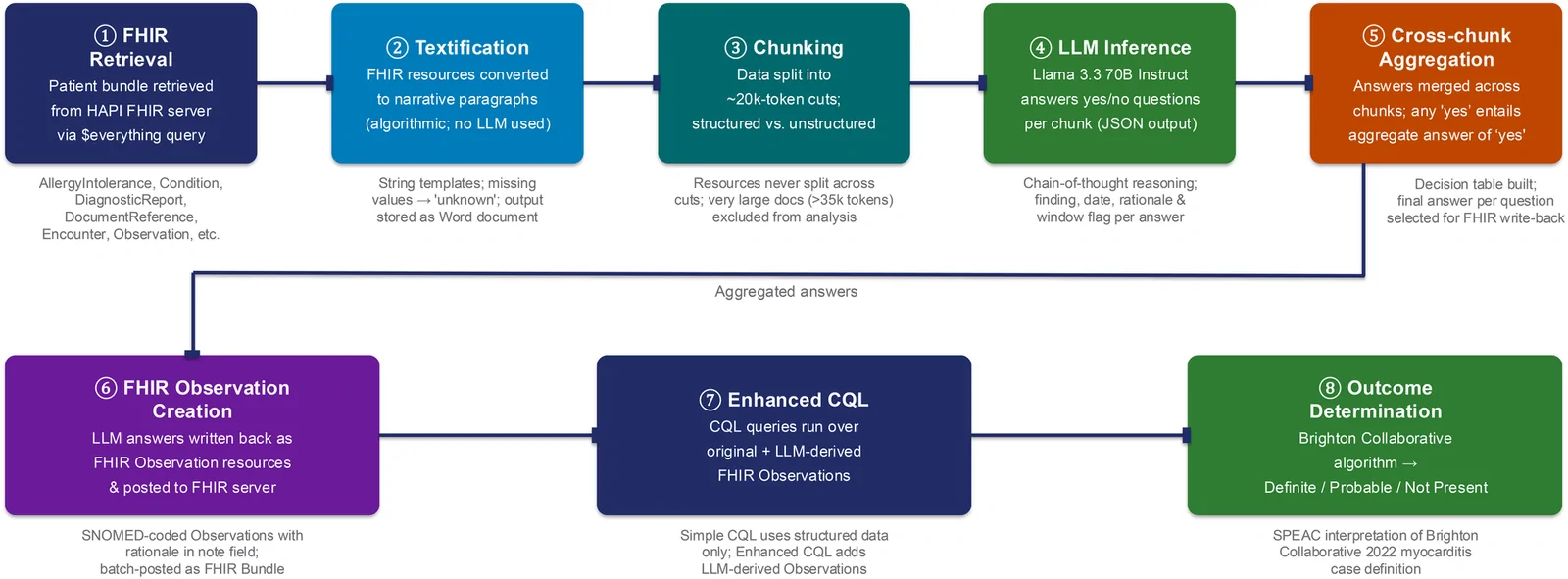
LLM feature extraction workflow.

With the provisional LLM answers to all key questions in hand, the decision table was used to create new structured data elements, specifically Observation resources, representing the LLM's answer to each of the key questions. For example, if the LLM determined for a specific patient that the clinical finding of “diaphoresis” was present, a FHIR Observation resource was created for that patient to represent this finding. Once all findings for a given patient had been processed, the newly created Observation resources for that patient were aggregated into a FHIR bundle. This bundle of LLM-derived Observation resources was posted back to the FHIR server and thus “enhanced” the original patient bundle with the newly created Observation resources. The LLM workflow is summarized in Figure 1.

### CQL determinations

Two CQL scripts were developed to find clinical information in the structured data only (herein called “simple CQL”) and the enhanced FHIR bundle (“enhanced CQL”) which contains clinical information from both structured and unstructured data. The simple CQL queried only cardiac symptoms, non-specific symptoms, and biomarker features from the Brighton Collaborative's 2022 myocarditis case definition (11) as these features have the potential to be in structured data, unlike other Brighton Collaborative myocarditis features such as imaging results that are likely only to be found in unstructured data (15, 16). Cardiac symptoms included chest pain, palpitations, dyspnea, diaphoresis, and sudden death. Non-specific symptoms included dizziness/syncope, abdominal pain, fatigue, edema, and cough. Cardiac and non-specific symptom value sets from the Value Set Authority Center (VSAC) were found for ICD-10-CM, ICD-9-CM, SNOMED-CT vocabularies (17). When required, ICD-9-CM codes were mapped from the ICD-10-CM codes in the values sets using the Centers for Medicare and Medicaid Services General Equivalence Mappings (18). Troponin and CK-MB value sets were developed by study investigators. Value sets are presented in Supplemental Material Tables S1–S7.

### Gold standard feature identification

Blinded to the LLMs output, three clinicians [internal medicine MD, emergency medicine DO, and nurse practitioner with a Doctor of Nursing Practice (DNP) degree] each independently reviewed a patient's clinical records and made a yes/no determination for each of the clinical features in the case definition and made a determination for presence or absence of the outcome (myocarditis present, yes/no). Clinicians followed a standardized protocol for patient review and discussed edge cases during the ‘optimization’ phase (discussed below). The majority answer across the three clinicians was considered the ‘gold-standard’ determination. Clinicians had access to a graphical user interface that displayed the FHIR resources and the ‘textified’ FHIR resource document utilized by the LLM within the secure FDA environment, but were blinded to the LLM answers.

### Statistical analysis

The study included both an ‘optimization’ and ‘test’ group of patients. The purpose of the optimization group was to 1) adjust LLM prompts and CQL logic to improve the automated phenotype determinations, 2) provide additional training to clinicians in reviewing cases, and 3) to work through unanticipated data edge cases or pipeline code bugs. The test set was used to assess performance of the LLM and CQL. The optimization set consisted of 10 patients, evenly split between health systems, and the test set consisted of 25 patients. For the test set, an independent clinician (MD/PhD) reviewed the textified data to filter out transplant patients (eight total transplant patients observed) prior to the three clinician reviewers adjudicating the myocarditis phenotype. Transplant patients were excluded due to clinician difficulty of reviewing thousands of pages of medical records for these complex cases and supposition that these potential myocarditis cases were likely not related to mRNA vaccine exposure. Discordances between the gold-standard and LLM for individual yes/no answers to Brighton Collaborative were reviewed by a fourth independent clinician (MD) and epidemiologist (PhD). The gold standard was corrected when the majority vote of the three validating clinicians was determined to be clearly wrong. In the test set, the majority vote of the three clinicians was determined to be wrong 23 times out of 600 questions (3.8%).

All statistical analyses were performed using Python, version 3.12.10. Binomial performance metrics included F1-score and Matthews Correlation Coefficient (MCC). Performance metrics were only calculated for question groups when there was at least one ‘yes’ gold-standard determination. Heuristic thresholds for moderate, good, and excellent performance were set for MCC and F1-scores *a priori.* For MCC, poor performance was <=0.4, moderate performance was >0.4–0.6, good performance >0.6–0.8, and excellent performance >0.8. For F1-score, poor performance was <=0.6, moderate performance was >0.6–0.75, good performance was >0.75–0.85, and excellent performance was >0.85. Because each patient contributed multiple questions, 95% confidence intervals were estimated using a patient-level nonparametric cluster bootstrap rather than methods assuming independence of individual questions. In each of 5,000 bootstrap replicates, 25 patients were sampled with replacement, with all 24 questions from each selected patient retained together. Question-level performance metrics, including F1-score and Matthews correlation coefficient, were recalculated for each replicate, and 95% confidence intervals were obtained from the bootstrap distribution (19, 20). For the trinomial outcome determinations, a linearly weighted Cohen's Kappa was used to test predictive performance, and 95% CI were calculated using the empirical distribution of the metric from 2,000 bootstrapped samples. Clinician reliability of agreement was quantified using the Fleiss’ Kappa statistic and percent of questions where all three clinicians provided the same answer.

### Sensitivity analyses

Two *post-hoc* sensitivity analyses were done to confirm the robustness of the study results. First, the performance of the LLM and CQL methodologies were recalculated using the test set without correcting the 23 gold-standards determined to be wrong by the fourth independent clinician and epidemiologist. This sensitivity analysis was performed to check the influence of correcting the gold-standard, which had a potential to bias the LLM and enhanced CQL results towards better performance. Second, the overall performance of the LLM and enhanced CQL across all questions was recalculated excluding the questions related to ECG abnormalities. This sensitivity analysis was performed due to the clinician agreement for ECG abnormalities being no better than chance; therefore, there is less confidence in our gold-standard answers to compare the LLM and enhanced CQL results.

### Human subjects considerations

Health systems provided FDA with identifiable patient record data as a public health surveillance activity as permitted by Federal regulation 45 CFR § 164.512(b)(1)(iii) (21). The work was conducted within the FDA's Sentinel BEST IM program as part of postmarket safety surveillance. The Office for Human Research Protections (OHRP) within the U.S. Department of Health and Human Services (HHS) has determined that studies performed under Sentinel are not subject to the OHRP-administered regulations at 45 CFR part 46 (22). Accordingly, written informed consent from participants—or from their legal guardians or next of kin—was not required under federal regulation and institutional policy (23).

## Results

After applying the initial inclusion criteria, there were 67 potential myocarditis patients. Patients were evenly split in the optimization set between the two health systems (Table 1); however, one health system had an abundance of transplant patients, which were subsequently excluded from the test set, resulting in health system 2 representing the majority of cases (96%). In the test set, most patients were vaccinated in 2021 and administered a vaccine manufactured by Pfizer. Further, the majority of test patients were between 12 and 29 years old, male, white, and not Hispanic or Latino.

**Table 1:** Characteristics of optimization and test patients.

Characteristic	Optimization (*n* = 10)	Test (*n* = 25)	All (*n* = 35)
Health system			
System 1	5 (50.0%)	1 (4.0%)	6 (17.1%)
System 2	5 (50.0%)	24 (96.0%)	29 (82.9%)
Vaccination year			
2021	2 (20.0%)	19 (76.0%)	21 (60.0%)
2022	2 (20.0%)	4 (16.0%)	6 (17.1%)
2023	4 (40.0%)	0 (0.0%)	4 (11.4%)
2024	1 (10.0%)	2 (8.0%)	3 (8.6%)
2025	1 (10.0%)	0 (0.0%)	1 (2.9%)
Vaccine manufacturer			
Moderna	1 (10.0%)	6 (24.0%)	7 (20.0%)
Pfizer	9 (90.0%)	19 (76.0%)	28 (80.0%)
Health system diagnosis			
Acute myocarditis	3 (30.0%)	7 (28.0%)	10 (28.6%)
Myocarditis	2 (20.0%)	17 (68.0%)	19 (54.3%)
Myocarditis, unspecified	5 (50.0%)	1 (4.0%)	6 (17.1%)
Age group (years)			
12–19	3 (30.0%)	9 (36.0%)	12 (34.3%)
20–29	0 (0.0%)	7 (28.0%)	7 (20.0%)
30–39	0 (0.0%)	2 (8.0%)	2 (5.7%)
40–49	1 (10.0%)	1 (4.0%)	2 (5.7%)
50–59	2 (20.0%)	2 (8.0%)	4 (11.4%)
60–69	4 (40.0%)	2 (8.0%)	6 (17.1%)
70–79	0 (0.0%)	2 (8.0%)	2 (5.7%)
Sex			
Female	2 (20.0%)	7 (28.0%)	9 (25.7%)
Male	8 (80.0%)	18 (72.0%)	26 (74.3%)
Race			
Asian	1 (10.0%)	2 (8.0%)	3 (8.6%)
Black or African American	3 (30.0%)	6 (24.0%)	9 (25.7%)
White	5 (50.0%)	15 (60.0%)	20 (57.1%)
Other race	1 (10.0%)	2 (8.0%)	3 (8.6%)
Ethnicity			
Hispanic or Latino	3 (30.0%)	2 (8.0%)	5 (14.3%)
Not Hispanic or Latino	7 (70.0%)	23 (92.0%)	30 (85.7%)

In the optimization and test set, a total of 240 and 600 yes/no questions, respectively, related to the myocarditis phenotype were answered by the clinicians. In the optimization set, the three clinicians unanimously agreed on the presence/absence of a clinical feature for 87.1% of the 240 yes/no questions, resulting in a Fleiss’ kappa statistic of 0.65. In the test set, the three clinicians unanimously agreed on the presence/absence of a clinical feature for 79.3% of the 600 yes/no questions, resulting in a Fleiss’ kappa statistic of 0.67. Clinician agreement differed by question type, as reflected by Fleiss’ kappa values: cardiac and non-specific symptoms (K = 0.81), cardiac biomarkers (K = 0.73), ECG abnormalities (K = 0.00), ECHO abnormalities (K = 0.57), CMR abnormalities (K = 0.88), and biopsy-confirmed myocardial inflammation (K = 0.79).

In the subset of questions the simple CQL was tasked to answer (i.e., symptom and biomarker questions), the simple CQL had moderate performance in the optimization set (*n* = 120) and poor performance in the test set (*n* = 300) (Table 2). Conversely, across all yes/no questions, the LLM performance rose from moderate in the optimization set (*n* = 240) to good in the test set (*n* = 600). For example, the F1-score was 0.64 (95% CI: 0.53–0.72) in the optimization set and 0.85 (95% CI: 0.81–0.88) in the test set. Following this trend, the enhanced CQL rose from moderate in the optimization set (*n* = 240) to excellent performance in the test set (*n* = 600). For example, the MCC was 0.58 (95% CI: 0.45–0.71) in the optimization set compared to 0.83 (95% CI: 0.77–0.88) in the test set. Confusion matrices are reported in Supplemental Material.

**Table 2:** Performance metrics (95% CI) of the three feature identification methods answering yes/no questions related to myocarditis in the test set.

Method	Set (number of questions)	MCC (95% CI)	F1-score (95% CI)
Simple CQL	Optimization (*n* = 120)	0.51 (0.39–0.69)	0.47 (0.31–0.67)
Simple CQL	Test (*n* = 300)	0.36 (0.31–0.41)	0.33 (0.27–0.39)
LLM	Optimization (*n* = 240)	0.59 (0.51–0.68)	0.64 (0.53–0.72)
LLM	Test (*n* = 600)	0.78 (0.73–0.83)	0.85 (0.81–0.88)
Enhanced CQL	Optimization (*n* = 240)	0.58 (0.45–0.71)	0.64 (0.51–0.76)
Enhanced CQL	Test (*n* = 600)	0.83 (0.77–0.88)	0.88 (0.84–0.92)

When the results were broken down to the different question groupings, the overall simple CQL poor performance can be attributed to the logic being unable to correctly identify cardiac and non-specific symptom features, where the MCC was 0.08 (95% CI: 0.00–0.15) and F1-score was 0.02 (95% CI: 0.00–0.07) in the test set (Table 3). However, the simple CQL achieved excellent performance in identifying elevated cardiac biomarkers of troponin I/T and CK-MB with an MCC of 0.83 (95% CI: 0.72–0.94) and F1-score was 0.88 (95% CI: 0.78–0.96) in the test set. The LLM had excellent performance in identifying symptoms, echocardiogram (ECHO) abnormalities, and cardiac magnetic resonance (CMR) abnormalities, but weaker performance in the case of cardiac biomarkers and electrocardiogram (ECG) abnormalities. The enhanced CQL had similar results to the LLM; however, the addition of structured laboratory data helped to improve enhanced CQL biomarker performance to excellent. Results are broken down by each individual question in the Supplemental Material but corroborate these general trends.

**Table 3:** Performance metrics (95% CI) of the three feature identification methods answering yes/no questions grouped by clinical concepts related to myocarditis in the test set. NC = not calculated due to simple CQL not tasked with identifying imaging abnormalities.

Grouping	Simple CQL		LLM		Enhanced CQL	
	MCC (95% CI)	F1-score (95% CI)	MCC (95% CI)	F1-score (95% CI)	MCC (95% CI)	F1-score (95% CI)
Cardiac and non-specific symptoms	0.08 (0.00–0.15)	0.02 (0.00–0.07)	0.85 (0.76–0.92)	0.90 (0.84–0.95)	0.86 (0.78–0.93)	0.91 (0.85–0.95)
Cardiac biomarkers	0.83 (0.72–0.94)	0.88 (0.78–0.96)	0.54 (0.46–0.61)	0.72 (0.67–0.77)	0.83 (0.72–0.94)	0.88 (0.78–0.96)
ECG abnormalities	NC	NC	0.68 (0.53–0.83)	0.74 (0.56–0.87)	0.68 (0.53–0.83)	0.74 (0.56–0.87)
ECHO abnormalities	NC	NC	0.86 (0.73–0.95)	0.90 (0.79–0.97)	0.86 (0.73–0.95)	0.90 (0.79–0.97)
CMR abnormalities	NC	NC	0.93 (0.70–1.00)	0.94 (0.67–1.00)	0.93 (0.70–1.00)	0.94 (0.67–1.00)

The individual yes/no questions were algorithmically combined, per SPEAC, for an outcome determination. Both the LLM and enhanced CQL correctly classified 23 of the 25 patients into the gold-standard outcome determinations (Table 4). The Cohen's Kappa was 0.85 (95% CI: 0.60–1.00) for the LLM and 0.86 (95% CI: 0.66–1.00) for the enhanced CQL.

**Table 4:** Confusion matrices comparing LLM, enhanced CQL, and gold-standard outcome determinations in the test set.

Method	Outcome determination	Gold-standard		
		No	Probable	Definite
LLM	No	1	0	0
	Probable	0	7	0
	Definite	0	2	15
Enhanced CQL	No	1	1	0
	Probable	0	7	0
	Definite	0	1	15

In *post-hoc* sensitivity analyses, the central findings were confirmed. However, in our first sensitivity analysis, where the gold-standard was not corrected by the fourth clinician and epidemiologist, the performance of all three feature identification methods decreased, although the decrease in performance was less pronounced in the simple CQL (Supplementary Table S10). The MCC and F1-score of the LLM in the test set went down to 0.70 (0.64–0.75) and 0.78 (0.73–0.83), respectively. The MCC and F1-score of the LLM in the test set went down to 0.74 (0.67–0.80) and 0.81 (0.76–0.86), respectively. The largest decrease in performance when the final adjudication was removed occurred in the ECG abnormalities, where the MCC and F1-score decreased by approximately 0.32 and 0.26, respectively, from the main performance scores. In the second sensitivity analysis, when ECG abnormality questions were removed, due to a high level of clinician disagreement, the overall LLM and enhanced CQL performance increased slightly. For example, across all myocarditis phenotype questions except the ECG abnormality question (*n* = 525), the LLM and enhanced CQL had an MCC of 0.80 (0.74–0.85) and 0.86 (0.80–0.91) in the test set, respectively, which was approximately 0.02–0.03 higher than the MCCs across all questions in the test set (*n* = 600).

## Discussion

In this research, multiple methods were tested to identify clinical features related to adverse event monitoring within FHIR resources. Myocarditis after COVID-19 vaccination served as a use case to test the potential viability of leveraging LLMs in monitoring adverse events. The simple CQL, which relied on structured data only, performed well in identifying cardiac biomarkers but not cardiac or non-specific symptoms. The LLM, which analyzed structured and unstructured data, showed strong ability to aid in the identification of various types of clinical features including symptoms and imaging abnormalities, but exhibited weaker performance in correctly answering questions related to cardiac biomarkers. The enhanced CQL, which was aided by both structured queries and LLM-extracted clinical concepts, exhibited strong performance identifying all clinical features. This work is consistent with previous work reporting that LLMs are an effective tool for clinical feature extraction from unstructured notes (6–9).

The LLM's poor performance in identifying cardiac biomarkers above a certain threshold was due to the LLM either 1) confusing troponin T with troponin I, despite the prompt explicitly instructing the LLM to focus on the specific subunit within the troponin complex, or 2) assuming ‘troponin’ was a troponin I or T. The LLM likely confuses the clinical concepts of troponin, troponin I, and troponin T because the Llama3 model generates similar and nearly indistinguishable vector representations for the words “troponin,” “troponin T,” and “troponin I.” Additionally, these concepts are often used interchangeably by clinicians, which would be expected to limit discrimination through additional natural language context. More advanced LLMs may be able to differentiate between troponin I and T, but future work should consider if differentiating similar concepts is necessary. Uniform cardiac biomarker thresholds from the literature were used to minimize heterogeneity introduced by laboratory-specific reference intervals. Although local upper reference limits may capture assay-specific normal ranges, they can vary across institutions and over time for reasons unrelated to disease biology; therefore, a single clinically established cutoff was prioritized to ensure consistent classification across sites. Given the LLM's poor performance in identifying cardiac biomarkers was unrelated to the actual thresholds used, the central findings of this research would not change based on using laboratory-specific vs. universal thresholds.

The enhanced CQL workflow relied heavily on the LLM extractions to answer the majority of questions. However, there were some differences in the results. In some instances, the CQL was given an enhanced resource with evidence, but the CQL engine correctly identified that the evidence was outside the evidence window. In addition, the LLM had a hard time distinguishing troponin I from troponin T, but the CQL logic could directly use the structured data elements representing these findings to make the correct determination. The enhanced CQL approach in this study complements the work in utilizing federated learning with LLMs for adverse event detection (24), where CQL queries could be sent via FHIR Library resource and run on health systems that have extracted pertinent clinical content from unstructured data into their FHIR server.

The FHIR data, even when just considering the 42-day evidence window, was large and required clinicians to review a large volume of information, potentially exceeding what clinicians could reasonably process when searching for answers to the questions. For example, in certain cases, when the FHIR data was ‘textified’, the resulting Word document could reach a thousand pages or more. Whether reviewed in the ‘textified’ format or within a graphical user interface displaying the FHIR data, identifying the key clinical details associated with myocarditis and understanding them in the context of sometimes very complex clinical scenarios with multiple admissions and comorbidities can be challenging for human reviewers. This challenge may be reflected in lower Fleiss’ kappa values, and clinician agreement, for categories such as ECG and ECHO abnormalities, where interpretation and extraction can be more subjective and dependent on how the information is recorded. While real world data is a valuable resource for adverse event detection, provision of tools that reduce clinician cognitive load during review, is a logical next step. Such tools could include LLMs finding potential informative portions of the medical record for the clinician to focus their attention on for review (25).

Limitations of this study include having only two health systems in the sample, particularly where the test set was mainly based in one health system. The validation was performed in a held-out internal set of patients vs. an external validation performed on patients from a new health system. Variations in health systems may affect the generalizability of the extraction and identification results. The LLM utilized in this study, Llama3.3 70B Instruct, was the only available LLM within the FDA infrastructure at the time of the study but represents a model that is several iterations behind the current state-of-the-art LLMs available in the field. More advanced models may improve the LLM performance; however, recent research found Llama3 70B to be one of the better performing LLMs for entity extraction from unstructured and semi-structured electronic health records (26). Our cardiac and non-specific symptom value sets used published VSAC code sets and, in some instances including abdominal pain, may have been too broad for the myocarditis phenotype. However, even using a broad code set that should increase symptom detection sensitivity, symptoms were rarely found in the structured data. This finding again highlights the limitations of relying solely on structured data for pharmacovigilance. Our study did not assess causality (i.e., myocarditis was caused by mRNA vaccination). Future BEST IM studies will incorporate synthesizing additional information to make causality assessments including the identification of differential diagnoses and more refined considerations of temporality. In this feasibility study, transplant patients were excluded from clinician adjudication in the test set because the disproportionately complex medical records of transplant patients made the clinician review time orders of magnitude longer. This exclusion likely improved specificity of attribution but may have introduced selection bias and reduced generalizability. Further, excluding transplant recipients may have increased the observed performance of the LLM by narrowing the clinical spectrum of cases under review. Finally, the final adjudication of gold-standards biased the performance of the feature extraction methods upward. The final adjudication was meant to understand the LLM reasoning when it made a mistake. However, occasionally, despite three independent reviews, the clinicians failed to find applicable information in the large amount of clinical information they were tasked to review.

To our knowledge, this is the first study to analyze FHIR resources with an LLM for adverse event detection. In this small feasibility study, the LLM excelled at identifying relevant pieces of clinical information in unstructured data and provides evidence that incorporating an LLM into pharmacovigilance practices using FHIR data is technically feasible. Future work could explore using LLMs to extract relevant information for a clinician to review (i.e., human-machine teaming). The LLM could either extract the applicable FHIR resources and/or create a succinct clinical summary for review by pharmacovigilance officers. LLM extractions of relevant evidence would also help alleviate the cognitive challenge of clinicians having to review large amounts of EHR data.

## Data Availability

The data analyzed in this study is subject to the following licenses/restrictions: Data is proprietary data from health systems. Requests to access these datasets should be directed to fgaspar@mitre.org.

## References

[1] Deady M Ezzeldin H Cook K Billings D Pizarro J Plotogea AA. The food and drug administration biologics effectiveness and safety initiative facilitates detection of vaccine administrations from unstructured data in medical records through natural language processing. Front Digit Health. (2021) 3:777905. 10.3389/fdgth.2021.777905 35005697 PMC8727347

[2] Deady M Duncan R Jones LD Sang A Goodness B Pandey A. Data quality and timeliness analysis for post-vaccination adverse event cases reported through healthcare data exchange to FDA BEST pilot platform. Front Public Health. (2024) 12:1379973. 10.3389/fpubh.2024.1379973 39040857 PMC11260708

[3] Deady M Duncan R Sonesen M Estiandan R Stimpert K Cho S. A computable phenotype algorithm for postvaccination myocarditis/pericarditis detection using real-world data: validation study. J Med Internet Res. (2024) 26:e54597. 10.2196/54597 39586081 PMC11629037

[4] Holdefer AA Pizarro J Saunders-Hastings P Beers J Sang A Hettinger AZ. Development of interoperable computable phenotype algorithms for adverse events of special interest to be used for biologics safety surveillance: validation study. JMIR Public Health Surveill. (2024) 10:e49811. 10.2196/49811 39008361 PMC11287092

[5] Zitu MM Owen D Manne A Wei P Li L. Large language models for adverse drug events: a clinical perspective. J Clin Med. (2025) 14(15):5490. 10.3390/jcm14155490 40807108 PMC12347610

[6] Boussina A Krishnamoorthy R Quintero K Joshi S Wardi G Pour H. Large language models for more efficient reporting of hospital quality measures. NEJM AI. (2024) 1(11):10.1056/aics2400420. 10.1056/aics2400420 PMC11658346 39703686

[7] Thakkar V Silverman GM Kc A Ingraham NE Jones EK King S. A comparative analysis of large language models versus traditional information extraction methods for real-world evidence of patient symptomatology in acute and post-acute sequelae of SARS-CoV-2. PLoS One. (2025) 20(5):e0323535. 10.1371/journal.pone.0323535 40373001 PMC12080813

[8] Reategui-Rivera CM Finkelstein J. Evaluation of the performance of a large language model to extract signs and symptoms from clinical notes. Stud Health Technol Inform. (2025) 323:71–5. 10.3233/SHTI250051 40200448

[9] Bunnell HT Reedy C Lorman V Jhaveri R Rivera-Sepulveda A Salamon KS. A natural language processing pipeline for identifying pediatric long COVID symptoms and functional impacts in freeform clinical notes: a RECOVER study. JAMIA Open. (2025) 8(5):ooaf089. 10.1093/jamiaopen/ooaf089 40918941 PMC12409404

[10] HL7 International. Da Vinci clinical data exchange (CDex). (2026). Available online at: https://build.fhir.org/ig/HL7/davinci-ecdx/ (Accessed January 5, 2026)

[11] Sexson Tejtel SK Munoz FM Al-Ammouri I Savorgnan F Guggilla RK Khuri-Bulos N. Myocarditis and pericarditis: case definition and guidelines for data collection, analysis, and presentation of immunization safety data. Vaccine. (2022) 40(10):1499–511. 10.1016/j.vaccine.2021.11.074 35105494

[12] Law B. AESI case definition companion guide for 2 Tier AESI—Myocarditis and pericarditis. Safety platform for emergency vaccines (SPEAC). (2022). Available online at: https://brightoncollaboration.org/wp-content/uploads/2023/06/SPEAC_D2.5.2.2_Myocarditis-companion-guide_codes-updated_BL_2022_May12.pdf

[13] American Board of Internal Medicine. ABIM laboratory test reference ranges. (2024). Available online at: https://www.abim.org/Media/bfijryql/laboratory-reference-ranges.pdf (Accessed January 24, 2025)

[14] Kurapati R Zubair M. Creatine kinase MB: diagnostic utility and limitations. In: StatPearls. Treasure Island (FL): StatPearls Publishing. (2025). Available online at: http://www.ncbi.nlm.nih.gov/books/NBK557591/ (Accessed January 24, 2025) 32491523

[15] Reichenpfader D Müller H Denecke K. A scoping review of large language model based approaches for information extraction from radiology reports. Npj Digit Med. (2024) 7(1):222. 10.1038/s41746-024-01219-0 39182008 PMC11344824

[16] Cai T Giannopoulos AA Yu S Kelil T Ripley B Kumamaru KK. Natural language processing technologies in radiology research and clinical applications. Radiographics. (2016) 36(1):176–91. 10.1148/rg.2016150080 26761536 PMC4734053

[17] National Library of Medicine. Value set authority center. Available online at: https://vsac.nlm.nih.gov/ (Accessed January 10, 2025)

[18] National Bureau of Economic Research. ICD-9-CM to and from ICD-10-CM and ICD-10-PCS crosswalk or general equivalence mappings. (2026). Available online at: https://www.nber.org/research/data/icd-9-cm-and-icd-10-cm-and-icd-10-pcs-crosswalk-or-general-equivalence-mappings (Accessed January 9, 2025)

[19] Carpenter J Bithell J. Bootstrap confidence intervals: when, which, what? A practical guide for medical statisticians. Statist Med. (2000) 19:1141–64. Available online at: https://onlinelibrary.wiley.com/doi/abs/10.1002/(SICI)1097-0258(20000515)19:9%3C1141::AID-SIM479%3E3.0.CO;2-F?msockid=37ac2494e23963482c883330e38f625b (Accessed July 29, 2026). 10.1002/(sici)1097-0258(20000515)19:9<1141::aid-sim479>3.0.co;2-f 10797513

[20] Field CA Welsh AH. Bootstrapping clustered data. J R Stat Soc Ser B Stat Methodol. (2007) 69(3):369–90. 10.1111/j.1467-9868.2007.00593.x

[21] CFR. 45 CFR 164.512—Uses and disclosures for which an authorization or opportunity to agree or object is not required. Available online at: https://www.ecfr.gov/current/title-45/part-164/section-164.512 (Accessed January 22, 2026)

[22] CFR. 45 CFR Part 46—Protection of human subjects. Available online at: https://www.ecfr.gov/current/title-45/part-46 (Accessed December 29, 2025)

[23] Gostin LO. National health information PrivacyRegulations under the health insurance portability and accountability act. JAMA. (2001) 285(23):3015–21. 10.1001/jama.285.23.3015 11410101

[24] Guo D Choo KKR. Applications of federated large language model for adverse drug reactions prediction: scoping review. J Med Internet Res. (2025) 27:e68291. 10.2196/68291 40921101 PMC12516295

[25] Rouhi AD Menon SV Ghanem YK Han JJ. Concordance of large language model recommendations with multidisciplinary heart team decisions in coronary revascularization and aortic valve intervention: a systematic review and pooled analysis. Cardiol Ther. (2026). 10.1007/s40119-026-00453-9 42141253 PMC13542011

[26] Ntinopoulos V Rodriguez Cetina Biefer H Tudorache I Papadopoulos N Odavic D Risteski P. Large language models for data extraction from unstructured and semi-structured electronic health records: a multiple model performance evaluation. BMJ Health Care Inform. (2025) 32(1):e101139. 10.1136/bmjhci-2024-101139 39832824 PMC11751965

