# Supplementary material for: LLM and CQL computable phenotypes for myocarditis adverse event feature and outcome determinations using patient FHIR data: a feasibility study
Source: Front Digit Health. 2026 Sep 8;8:1903755. doi: 10.3389/fdgth.2026.1903755 (PMC13593815; doi:10.3389/fdgth.2026.1903755)
Supplement: Supplementary file 1 [file Supplementaryfile1.docx]

**Supplementary Materials:**

**LLM and CQL Computable Phenotypes for Myocarditis Adverse Event Feature and Outcome Determinations using Patient FHIR Data: A Feasibility Study**

## Value Set Information

Table S1. mRNA value set

| **Code** | **Description** | **Vocabulary** |
| --- | --- | --- |
| 207 | COVID-19, mRNA, LNP-S, PF, 100 mcg/0.5mL dose or 50 mcg/0.25mL dose | CVX |
| 208 | COVID-19, mRNA, LNP-S, PF, 30 mcg/0.3 mL dose | CVX |
| 217 | COVID-19, mRNA, LNP-S, PF, 30 mcg/0.3 mL dose, tris-sucrose | CVX |
| 218 | COVID-19, mRNA, LNP-S, PF, 10 mcg/0.2 mL dose, tris-sucrose | CVX |
| 219 | COVID-19, mRNA, LNP-S, PF, 3 mcg/0.2 mL dose, tris-sucrose | CVX |
| 221 | COVID-19, mRNA, LNP-S, PF, 50 mcg/0.5 mL dose | CVX |
| 228 | COVID-19, mRNA, LNP-S, PF, pediatric 25 mcg/0.25 mL dose | CVX |
| 229 | COVID-19, mRNA, LNP-S, bivalent, PF, 50 mcg/0.5 mL or 25mcg/0.25 mL dose | CVX |
| 230 | COVID-19, mRNA, LNP-S, bivalent, PF, 10 mcg/0.2 mL | CVX |
| 300 | COVID-19, mRNA, LNP-S, bivalent, PF, 30 mcg/0.3 mL dose | CVX |
| 301 | COVID-19, mRNA, LNP-S, bivalent, PF, 10 mcg/0.2 mL dose | CVX |
| 302 | COVID-19, mRNA, LNP-S, bivalent, PF, 3 mcg/0.2 mL dose | CVX |
| 308 | COVID-19, mRNA, LNP-S, PF, tris-sucrose, 3 mcg/0.3 mL | CVX |
| 309 | COVID-19, mRNA, LNP-S, PF, tris-sucrose, 30 mcg/0.3 mL | CVX |
| 310 | COVID-19, mRNA, LNP-S, PF, tris-sucrose, 10 mcg/0.3 mL | CVX |
| 311 | COVID-19, mRNA, LNP-S, PF, 25 mcg/0.25 mL | CVX |
| 312 | COVID-19, mRNA, LNP-S, PF, 50 mcg/0.5 mL | CVX |
| 90683 | Respiratory syncytial virus vaccine, mRNA lipid nanoparticles, for intramuscular use | CPT |
| 91300 | Severe acute respiratory syndrome coronavirus 2 (SARS-CoV-2) (coronavirus disease [COVID-19]) vaccine, mRNA-LNP, spike protein, preservative free, 30 mcg/0.3 mL dosage, diluent reconstituted, for intramuscular use | CPT |
| 91301 | Severe acute respiratory syndrome coronavirus 2 (SARS-CoV-2) (coronavirus disease [COVID-19]) vaccine, mRNA-LNP, spike protein, preservative free, 100 mcg/0.5 mL dosage, for intramuscular use | CPT |
| 91305 | Severe acute respiratory syndrome coronavirus 2 (SARS-CoV-2) (coronavirus disease [COVID-19]) vaccine, mRNA-LNP, spike protein, preservative free, 30 mcg/0.3 mL dosage, tris-sucrose formulation, for intramuscular use | CPT |
| 91306 | Severe acute respiratory syndrome coronavirus 2 (SARS-CoV-2) (coronavirus disease [COVID-19]) vaccine, mRNA-LNP, spike protein, preservative free, 50 mcg/0.25 mL dosage, for intramuscular use | CPT |
| 91307 | Severe acute respiratory syndrome coronavirus 2 (SARS-CoV-2) (coronavirus disease [COVID-19]) vaccine, mRNA-LNP, spike protein, preservative free, 10 mcg/0.2 mL dosage, diluent reconstituted, tris-sucrose formulation, for intramuscular use | CPT |
| 91308 | Severe acute respiratory syndrome coronavirus 2 (SARS-CoV-2) (coronavirus disease [COVID-19]) vaccine, mRNA-LNP, spike protein, preservative free, 3 mcg/0.2 mL dosage, diluent reconstituted, tris-sucrose formulation, for intramuscular use | CPT |
| 91309 | Severe acute respiratory syndrome coronavirus 2 (SARS-CoV-2) (coronavirus disease [COVID-19]) vaccine, mRNA-LNP, spike protein, preservative free, 50 mcg/0.5 mL dosage, for intramuscular use | CPT |
| 91311 | Severe acute respiratory syndrome coronavirus 2 (SARS-CoV-2) (coronavirus disease [COVID-19]) vaccine, mRNA-LNP, spike protein, preservative free, 25 mcg/0.25 mL dosage, for intramuscular use | CPT |
| 91312 | Severe acute respiratory syndrome coronavirus 2 (SARS-CoV-2) (coronavirus disease [COVID-19]) vaccine, mRNA-LNP, bivalent spike protein, preservative free, 30 mcg/0.3 mL dosage, tris-sucrose formulation, for intramuscular use | CPT |
| 91313 | Severe acute respiratory syndrome coronavirus 2 (SARS-CoV-2) (coronavirus disease [COVID-19]) vaccine, mRNA-LNP, spike protein, bivalent, preservative free, 50 mcg/0.5 mL dosage, for intramuscular use | CPT |
| 91314 | Severe acute respiratory syndrome coronavirus 2 (SARS-CoV-2) (coronavirus disease [COVID-19]) vaccine, mRNA-LNP, spike protein, bivalent, preservative free, 25 mcg/0.25 mL dosage, for intramuscular use | CPT |
| 91315 | Severe acute respiratory syndrome coronavirus 2 (SARS-CoV-2) (coronavirus disease [COVID-19]) vaccine, mRNA-LNP, bivalent spike protein, preservative free, 10 mcg/0.2 mL dosage, diluent reconstituted, tris-sucrose formulation, for intramuscular use | CPT |
| 91316 | Severe acute respiratory syndrome coronavirus 2 (SARS-CoV-2) (coronavirus disease [COVID-19]) vaccine, mRNA-LNP, spike protein, bivalent, preservative free, 10 mcg/0.2 mL dosage, for intramuscular use | CPT |
| 91317 | Severe acute respiratory syndrome coronavirus 2 (SARS-CoV-2) (coronavirus disease [COVID-19]) vaccine, mRNA-LNP, bivalent spike protein, preservative free, 3 mcg/0.2 mL dosage, diluent reconstituted, tris-sucrose formulation, for intramuscular use | CPT |
| 91318 | Severe acute respiratory syndrome coronavirus 2 (SARS-CoV-2) (coronavirus disease [COVID-19]) vaccine, mRNA-LNP, spike protein, 3 mcg/0.3 mL dosage, tris-sucrose formulation, for intramuscular use | CPT |
| 91319 | Severe acute respiratory syndrome coronavirus 2 (SARS-CoV-2) (coronavirus disease [COVID-19]) vaccine, mRNA-LNP, spike protein, 10 mcg/0.3 mL dosage, tris-sucrose formulation, for intramuscular use | CPT |
| 91320 | Severe acute respiratory syndrome coronavirus 2 (SARS-CoV-2) (coronavirus disease [COVID-19]) vaccine, mRNA-LNP, spike protein, 30 mcg/0.3 mL dosage, tris-sucrose formulation, for intramuscular use | CPT |
| 91321 | Severe acute respiratory syndrome coronavirus 2 (SARS-CoV-2) (coronavirus disease [COVID-19]) vaccine, mRNA-LNP, 25 mcg/0.25 mL dosage, for intramuscular use | CPT |
| 91322 | Severe acute respiratory syndrome coronavirus 2 (SARS-CoV-2) (coronavirus disease [COVID-19]) vaccine, mRNA-LNP, 50 mcg/0.5 mL dosage, for intramuscular use | CPT |

Table S2. Myocarditis value set

| **Code** | **Description** | **Vocabulary** | **Etiology Implied** |
| --- | --- | --- | --- |
| I51.4 | Myocarditis, unspecified | ICD-10-CM | No |
| I01.2 | Acute rheumatic myocarditis | ICD-10-CM | Yes |
| I40.9 | Acute myocarditis, unspecified | ICD-10-CM | No |
| I09.0 | Rheumatic myocarditis | ICD-10-CM | Yes |
| I40.1 | Isolated myocarditis | ICD-10-CM | No |
| B33.22 | Viral myocarditis | ICD-10-CM | Yes |
| I40.0 | Infective myocarditis | ICD-10-CM | Yes |
| A39.52 | Meningococcal myocarditis | ICD-10-CM | Yes |
| A36.81 | Diphtheritic cardiomyopathy (Diphtheritic myocarditis) | ICD-10-CM | Yes |
| I40.8 | Other acute myocarditis | ICD-10-CM | No |
| B58.81 | Toxoplasma myocarditis | ICD-10-CM | Yes |
| A52.06 | Other syphilitic heart involvement (Syphylitic myocarditis and Syphylitic pericarditis) | ICD-10-CM | Yes |
| B26.82 | Mumps myocarditis | ICD-10-CM | Yes |
| D86.85 | Sarcoid myocarditis | ICD-10-CM | Yes |
| J11.82 | Influenza due to unidentified influenza virus with myocarditis | ICD-10-CM | Yes |
| I41 | Myocarditis in diseases classified elsewhere | ICD-10-CM | No |
| A54.83 | Gonococcal heart infection (Gonococcal myocarditis and Gonococcal pericarditis) | ICD-10-CM | Yes |
| A18.84 | Tuberculosis of heart (Tuberculosis myocarditis and Tuberculosis pericarditis) | ICD-10-CM | Yes |
| A38.1 | Scarlet fever with myocarditis | ICD-10-CM | Yes |
| J10.82 | Influenza due to other identified influenza virus with myocarditis | ICD-10-CM | Yes |
| A01.02 | Typhoid fever with heart involvement (Typhoid myocarditis) | ICD-10-CM | Yes |
| B57.0 | Acute Chagas' disease with heart involvement (Acute Chagas' disease with myocarditis) | ICD-10-CM | Yes |
| B57.2 | Chagas' disease (chronic) with heart involvement (Chagas' disease (chronic) with myocarditis) | ICD-10-CM | No |
| J09.X9 | Influenza due to identified novel influenza A virus with other manifestations (Influenza due to identified novel influenza A virus with myocarditis) | ICD-10-CM | Yes |
| 50920009 | Myocarditis | SNOMED-CT | No |
| 194709000 | Acute rheumatic myocarditis | SNOMED-CT | Yes |
| 46701001 | Acute myocarditis | SNOMED-CT | No |
| 195136004 | Rheumatoid myocarditis | SNOMED-CT | Yes |
| 91025000 | Idiopathic myocarditis | SNOMED-CT | No |
| 266238009 | Isolated (Fiedler's) myocarditis | SNOMED-CT | No |
| 89141000 | Viral myocarditis | SNOMED-CT | Yes |
| 22653005 | Myocarditis due to infectious agent | SNOMED-CT | Yes |
| 64043005 | Bacterial myocarditis | SNOMED-CT | Yes |
| 60812006 | Giant cell myocarditis | SNOMED-CT | No |
| 91468009 | Meningococcal myocarditis | SNOMED-CT | Yes |
| 26117009 | Diphtheritic myocarditis | SNOMED-CT | Yes |
| 31993003 | Toxic myocarditis | SNOMED-CT | No |
| 4082005 | Syphilitic myocarditis | SNOMED-CT | Yes |
| 187195003 | Toxoplasma myocarditis | SNOMED-CT | Yes |
| 76534005 | Myocarditis due to acquired toxoplasmosis | SNOMED-CT | Yes |
| 37925008 | Interstitial myocarditis | SNOMED-CT | No |
| 30496006 | Dilated cardiomyopathy due to viral myocarditis | SNOMED-CT | Yes |
| 37217002 | Coxsackie myocarditis | SNOMED-CT | Yes |
| 45093008 | Chronic interstitial myocarditis | SNOMED-CT | No |
| 194950003 | Acute myocarditis - meningococcal | SNOMED-CT | Yes |
| 63462008 | Mumps myocarditis | SNOMED-CT | Yes |
| 279001 | Senile myocarditis | SNOMED-CT | Yes |
| 61012002 | Chronic rheumatic myocarditis | SNOMED-CT | No |
| 195033009 | Sarcoid heart muscle disease | SNOMED-CT | Yes |
| 451241000124108 | Chronic myocarditis | SNOMED-CT | No |
| 78046005 | Myocarditis caused by influenza virus | SNOMED-CT | Yes |
| 74918002 | Measles myocarditis | SNOMED-CT | Yes |
| 64190005 | Rubella myocarditis | SNOMED-CT | Yes |
| 427372006 | Eosinophilic myocarditis | SNOMED-CT | No |
| 488007 | Fibroid myocarditis | SNOMED-CT | No |
| 194957000 | Septic myocarditis - staphylococcal | SNOMED-CT | Yes |
| 194958005 | Septic myocarditis - streptococcal | SNOMED-CT | Yes |
| 194956009 | Septic myocarditis - pneumococcal | SNOMED-CT | Yes |
| 194949003 | Acute myocarditis - tuberculous | SNOMED-CT | Yes |
| 194948006 | Acute myocarditis - toxoplasmosis | SNOMED-CT | Yes |
| 194947001 | Acute syphilitic myocarditis | SNOMED-CT | Yes |
| 55482007 | Isolated diffuse granulomatous myocarditis | SNOMED-CT | No |
| 421272004 | Subacute myocarditis with AIDS (acquired immunodeficiency syndrome) | SNOMED-CT | Yes |
| 1052287002 | Gonococcal myocarditis | SNOMED-CT | Yes |
| 65718001 | Adenoviral myocarditis | SNOMED-CT | Yes |
| 8916007 | Protozoan myocarditis | SNOMED-CT | Yes |
| 233868005 | Fungal myocarditis | SNOMED-CT | Yes |
| 233869002 | Parasitic myocarditis | SNOMED-CT | Yes |
| 18484008 | Subacute interstitial myocarditis | SNOMED-CT | No |
| 233867000 | Q fever myocarditis | SNOMED-CT | Yes |
| 194946005 | Acute myocarditis - influenza | SNOMED-CT | Yes |
| 194944008 | Acute myocarditis - coxsackie | SNOMED-CT | Yes |
| 194945009 | Acute myocarditis - diphtheritic | SNOMED-CT | Yes |
| 421929001 | Myocarditis with AIDS (acquired immunodeficiency syndrome) | SNOMED-CT | Yes |
| 88782004 | Myocarditis caused by radiation | SNOMED-CT | Yes |
| 72527006 | Myocarditis caused by drug | SNOMED-CT | Yes |
| 194942007 | Acute myocarditis associated with another disorder | SNOMED-CT | No |
| 194943002 | Acute aseptic myocarditis of the newborn | SNOMED-CT | No |
| 55087008 | Myocarditis due to chemical agent | SNOMED-CT | Yes |
| 8676001 | Myocarditis due to hypersensitivity state | SNOMED-CT | Yes |
| 79096004 | Myocarditis caused by physical agent | SNOMED-CT | Yes |
| 427443009 | Lymphocytic myocarditis | SNOMED-CT | No |
| 472707009 | Fetal myocarditis | SNOMED-CT | No |
| 713318009 | Myocarditis co-occurrent with human immunodeficiency virus infection | SNOMED-CT | Yes |
| 460345001 | Myocarditis caused by echovirus | SNOMED-CT | Yes |
| 460317007 | Myocarditis caused by Rickettsia | SNOMED-CT | Yes |
| 460338001 | Myocarditis caused by Aspergillus | SNOMED-CT | Yes |
| 460352004 | Myocarditis caused by Spirochaetales | SNOMED-CT | Yes |
| 460620006 | Myocarditis caused by Borrelia | SNOMED-CT | Yes |
| 460329003 | Myocarditis caused by Candida | SNOMED-CT | Yes |
| 40532006 | Dilated cardiomyopathy due to protozoan myocarditis | SNOMED-CT | Yes |
| 44630002 | Dilated cardiomyopathy due to fungal myocarditis | SNOMED-CT | Yes |
| 54830008 | Dilated cardiomyopathy due to bacterial myocarditis | SNOMED-CT | Yes |
| 703274008 | Heart failure with reduced ejection fraction due to myocarditis | SNOMED-CT | No |
| 1204204003 | Left ventricular failure with normal ejection fraction due to myocarditis | SNOMED-CT | No |
| 681211000119101 | History of myocarditis | SNOMED-CT | No |
| 413933004 | Coxsackie myocarditis of newborn | SNOMED-CT | Yes |
| 871587002 | Myocarditis due to Chaga disease | SNOMED-CT | Yes |
| 871640001 | Myocarditis due to autoimmune disease | SNOMED-CT | Yes |
| 870301007 | Myocarditis due to childhood type dermatomyositis | SNOMED-CT | Yes |
| 880012008 | Myocarditis due to Kawasaki disease | SNOMED-CT | Yes |
| 1089931000119105 | Myocarditis due to scarlet fever | SNOMED-CT | Yes |
| 1240531000000103 | Myocarditis due to COVID-19 | SNOMED-CT | Yes |
| 142951000119106 | Myocarditis caused by Influenza A virus subtype H1N1 | SNOMED-CT | Yes |
| 472811009 | Cardiovascular abnormality due to infective myocarditis | SNOMED-CT | Yes |
| 10629351000119108 | Myocarditis caused by Influenza A virus | SNOMED-CT | Yes |
| 1153452008 | Dilated cardiomyopathy due to parasitic myocarditis | SNOMED-CT | Yes |
| 1208897003 | Myocarditis due to idiopathic hypereosinophilic syndrome | SNOMED-CT | Yes |
| 471841009 | Inflammatory cardiomyopathy | SNOMED-CT | No |
| 30328003 | Acute myoendocarditis | SNOMED-CT | No |
| 69589006 | Subacute myoendocarditis | SNOMED-CT | No |
| 1204460007 | Myocardial dysfunction with sepsis | SNOMED-CT | No |

Table S3. Symptom value set object identifiers (OIDS) from the Value Set Authority Center

| **Clinical concept** | **Value set source** |
| --- | --- |
| Chest pain | 2.16.840.1.113883.3.526.3.1526 |
| Abdominal pain | 2.16.840.1.113762.1.4.1146.680, 2.16.840.1.113762.1.4.1146.681 |
| Cough | 2.16.840.1.113762.1.4.1146.90, 2.16.840.1.113762.1.4.1146.91 |
| Diaphoresis | 2.16.840.1.113762.1.4.1146.2437,  2.16.840.1.113762.1.4.1146.2436 |
| Dizziness | 2.16.840.1.113883.3.3616.200.110.102.6315, 2.16.840.1.113883.3.3616.200.110.102.3223 |
| Dyspnea | 2.16.840.1.113762.1.4.1182.47 |
| Fatigue | 2.16.840.1.113762.1.4.1146.862, 2.16.840.1.113762.1.4.1146.860 |
| Palpitations | 2.16.840.1.113883.3.3616.200.110.102.3224 |
| Peripheral edema | 2.16.840.1.113762.1.4.1078.894 |

Table S4. Cardiac and non-specific symptom value set

| **Code** | **Description** | **Vocabulary** | **Symptom** |
| --- | --- | --- | --- |
| R10.0 | Acute abdomen | ICD-10-CM | Abdominal pain |
| R10.10 | Upper abdominal pain, unspecified | ICD-10-CM | Abdominal pain |
| R10.11 | Right upper quadrant pain | ICD-10-CM | Abdominal pain |
| R10.12 | Left upper quadrant pain | ICD-10-CM | Abdominal pain |
| R10.13 | Epigastric pain | ICD-10-CM | Abdominal pain |
| R10.2 | Pelvic and perineal pain | ICD-10-CM | Abdominal pain |
| R10.30 | Lower abdominal pain, unspecified | ICD-10-CM | Abdominal pain |
| R10.31 | Right lower quadrant pain | ICD-10-CM | Abdominal pain |
| R10.32 | Left lower quadrant pain | ICD-10-CM | Abdominal pain |
| R10.33 | Periumbilical pain | ICD-10-CM | Abdominal pain |
| R10.811 | Right upper quadrant abdominal tenderness | ICD-10-CM | Abdominal pain |
| R10.812 | Left upper quadrant abdominal tenderness | ICD-10-CM | Abdominal pain |
| R10.813 | Right lower quadrant abdominal tenderness | ICD-10-CM | Abdominal pain |
| R10.814 | Left lower quadrant abdominal tenderness | ICD-10-CM | Abdominal pain |
| R10.815 | Periumbilic abdominal tenderness | ICD-10-CM | Abdominal pain |
| R10.816 | Epigastric abdominal tenderness | ICD-10-CM | Abdominal pain |
| R10.817 | Generalized abdominal tenderness | ICD-10-CM | Abdominal pain |
| R10.819 | Abdominal tenderness, unspecified site | ICD-10-CM | Abdominal pain |
| R10.821 | Right upper quadrant rebound abdominal tenderness | ICD-10-CM | Abdominal pain |
| R10.822 | Left upper quadrant rebound abdominal tenderness | ICD-10-CM | Abdominal pain |
| R10.823 | Right lower quadrant rebound abdominal tenderness | ICD-10-CM | Abdominal pain |
| R10.824 | Left lower quadrant rebound abdominal tenderness | ICD-10-CM | Abdominal pain |
| R10.825 | Periumbilic rebound abdominal tenderness | ICD-10-CM | Abdominal pain |
| R10.826 | Epigastric rebound abdominal tenderness | ICD-10-CM | Abdominal pain |
| R10.827 | Generalized rebound abdominal tenderness | ICD-10-CM | Abdominal pain |
| R10.829 | Rebound abdominal tenderness, unspecified site | ICD-10-CM | Abdominal pain |
| R10.84 | Generalized abdominal pain | ICD-10-CM | Abdominal pain |
| R10.9 | Unspecified abdominal pain | ICD-10-CM | Abdominal pain |
| 789.00 | Abdominal pain, unspecified site | ICD-9-CM | Abdominal pain |
| 789.09 | Abdominal pain, other specified site | ICD-9-CM | Abdominal pain |
| 789.01 | Abdominal pain, right upper quadrant | ICD-9-CM | Abdominal pain |
| 789.02 | Abdominal pain, left upper quadrant | ICD-9-CM | Abdominal pain |
| 789.06 | Abdominal pain, epigastric | ICD-9-CM | Abdominal pain |
| 789.03 | Abdominal pain, right lower quadrant | ICD-9-CM | Abdominal pain |
| 789.04 | Abdominal pain, left lower quadrant | ICD-9-CM | Abdominal pain |
| 789.05 | Abdominal pain, periumbilic | ICD-9-CM | Abdominal pain |
| 789.61 | Abdominal tenderness, right upper quadrant | ICD-9-CM | Abdominal pain |
| 789.62 | Abdominal tenderness, left upper quadrant | ICD-9-CM | Abdominal pain |
| 789.63 | Abdominal tenderness, right lower quadrant | ICD-9-CM | Abdominal pain |
| 789.64 | Abdominal tenderness, left lower quadrant | ICD-9-CM | Abdominal pain |
| 789.65 | Abdominal tenderness, periumbilic | ICD-9-CM | Abdominal pain |
| 789.66 | Abdominal tenderness, epigastric | ICD-9-CM | Abdominal pain |
| 789.67 | Abdominal tenderness, generalized | ICD-9-CM | Abdominal pain |
| 789.60 | Abdominal tenderness, unspecified site | ICD-9-CM | Abdominal pain |
| 789.69 | Abdominal tenderness, other specified site | ICD-9-CM | Abdominal pain |
| 789.07 | Abdominal pain, generalized | ICD-9-CM | Abdominal pain |
| 102613000 | Localized abdominal pain (finding) | SNOMED-CT | Abdominal pain |
| 102614006 | Generalized abdominal pain (finding) | SNOMED-CT | Abdominal pain |
| 1119211005 | Tenderness of left abdominal lumbar region (finding) | SNOMED-CT | Abdominal pain |
| 1119212003 | Tenderness of right abdominal lumbar region (finding) | SNOMED-CT | Abdominal pain |
| 1119217009 | Pain in right abdominal lumbar region (finding) | SNOMED-CT | Abdominal pain |
| 1119218004 | Pain in left abdominal lumbar region (finding) | SNOMED-CT | Abdominal pain |
| 111985007 | Chronic abdominal pain (finding) | SNOMED-CT | Abdominal pain |
| 1157014003 | Rebound tenderness of left upper quadrant of abdomen (finding) | SNOMED-CT | Abdominal pain |
| 1157015002 | Rebound tenderness of left lower quadrant of abdomen (finding) | SNOMED-CT | Abdominal pain |
| 1157016001 | Rebound tenderness of right lower quadrant of abdomen (finding) | SNOMED-CT | Abdominal pain |
| 116290004 | Acute abdominal pain (finding) | SNOMED-CT | Abdominal pain |
| 14700001000004102 | Intractable abdominal pain (finding) | SNOMED-CT | Abdominal pain |
| 162038003 | Non-colicky abdominal pain (finding) | SNOMED-CT | Abdominal pain |
| 162042000 | Abdominal wall pain (finding) | SNOMED-CT | Abdominal pain |
| 162046002 | Central abdominal pain (finding) | SNOMED-CT | Abdominal pain |
| 21522001 | Abdominal pain (finding) | SNOMED-CT | Abdominal pain |
| 235841007 | Chronic nonspecific abdominal pain (finding) | SNOMED-CT | Abdominal pain |
| 247362001 | Pain on abdominal wall movement (finding) | SNOMED-CT | Abdominal pain |
| 249545003 | Abdominal guarding (finding) | SNOMED-CT | Abdominal pain |
| 271858001 | Recurrent acute abdominal pain (finding) | SNOMED-CT | Abdominal pain |
| 28221000119103 | Abdominal muscle pain (finding) | SNOMED-CT | Abdominal pain |
| 285387005 | Left sided abdominal pain (finding) | SNOMED-CT | Abdominal pain |
| 285388000 | Right sided abdominal pain (finding) | SNOMED-CT | Abdominal pain |
| 301406006 | Tenderness of central region of abdomen (finding) | SNOMED-CT | Abdominal pain |
| 301715003 | Left upper quadrant pain (finding) | SNOMED-CT | Abdominal pain |
| 301716002 | Left lower quadrant pain (finding) | SNOMED-CT | Abdominal pain |
| 301717006 | Right upper quadrant pain (finding) | SNOMED-CT | Abdominal pain |
| 301754002 | Right lower quadrant pain (finding) | SNOMED-CT | Abdominal pain |
| 304542004 | Nonspecific abdominal pain (finding) | SNOMED-CT | Abdominal pain |
| 314212008 | Abdominal pain - cause unknown (finding) | SNOMED-CT | Abdominal pain |
| 35611005 | Rebound tenderness (finding) | SNOMED-CT | Abdominal pain |
| 371102005 | Generalized colicky abdominal pain (finding) | SNOMED-CT | Abdominal pain |
| 43364001 | Abdominal discomfort (finding) | SNOMED-CT | Abdominal pain |
| 43478001 | Abdominal tenderness (finding) | SNOMED-CT | Abdominal pain |
| 438506002 | Visceral abdominal pain (finding) | SNOMED-CT | Abdominal pain |
| 439469002 | Recurrent abdominal pain (finding) | SNOMED-CT | Abdominal pain |
| 439774009 | Tenderness of left lower quadrant of abdomen (finding) | SNOMED-CT | Abdominal pain |
| 440220007 | Generalized abdominal tenderness (finding) | SNOMED-CT | Abdominal pain |
| 443503005 | Periumbilical pain (finding) | SNOMED-CT | Abdominal pain |
| 444746004 | Acute exacerbation of chronic abdominal pain (finding) | SNOMED-CT | Abdominal pain |
| 448265002 | Tenderness of right upper quadrant of abdomen (finding) | SNOMED-CT | Abdominal pain |
| 448660000 | Tenderness of left upper quadrant of abdomen (finding) | SNOMED-CT | Abdominal pain |
| 448661001 | Tenderness of right lower quadrant of abdomen (finding) | SNOMED-CT | Abdominal pain |
| 449890002 | Functional abdominal pain syndrome (disorder) | SNOMED-CT | Abdominal pain |
| 45979003 | Abdominal wind pain (finding) | SNOMED-CT | Abdominal pain |
| 54586004 | Lower abdominal pain (finding) | SNOMED-CT | Abdominal pain |
| 60043000 | Midabdominal crampy pain (finding) | SNOMED-CT | Abdominal pain |
| 703100191000119101 | Rebound tenderness of right upper quadrant of abdomen (finding) | SNOMED-CT | Abdominal pain |
| 707597009 | Continuous abdominal pain of left lower quadrant (finding) | SNOMED-CT | Abdominal pain |
| 71850005 | Abdominal pain worse on motion (finding) | SNOMED-CT | Abdominal pain |
| 74704000 | Abdominal pain through to back (finding) | SNOMED-CT | Abdominal pain |
| 82648006 | Abdominal involuntary guarding (finding) | SNOMED-CT | Abdominal pain |
| 82708005 | Abdominal voluntary guarding (finding) | SNOMED-CT | Abdominal pain |
| 83132003 | Upper abdominal pain (finding) | SNOMED-CT | Abdominal pain |
| 838411007 | Pain in abdominal region on palpation (finding) | SNOMED-CT | Abdominal pain |
| 9209005 | Acute abdomen (disorder) | SNOMED-CT | Abdominal pain |
| 9991008 | Abdominal colic (finding) | SNOMED-CT | Abdominal pain |
| R07.1 | Chest pain on breathing | ICD-10-CM | Chest pain |
| R07.2 | Precordial pain | ICD-10-CM | Chest pain |
| R07.81 | Pleurodynia | ICD-10-CM | Chest pain |
| R07.82 | Intercostal pain | ICD-10-CM | Chest pain |
| R07.89 | Other chest pain | ICD-10-CM | Chest pain |
| R07.9 | Chest pain, unspecified | ICD-10-CM | Chest pain |
| 786.52 | Painful respiration | ICD-9-CM | Chest pain |
| 786.51 | Precordial pain | ICD-9-CM | Chest pain |
| 786.59 | Other chest pain | ICD-9-CM | Chest pain |
| 786.50 | Chest pain, unspecified | ICD-9-CM | Chest pain |
| 10000006 | Radiating chest pain (finding) | SNOMED-CT | Chest pain |
| 102587001 | Acute chest pain (finding) | SNOMED-CT | Chest pain |
| 102588006 | Chest wall pain (finding) | SNOMED-CT | Chest pain |
| 102589003 | Atypical chest pain (finding) | SNOMED-CT | Chest pain |
| 102591006 | Tenderness of chest wall (finding) | SNOMED-CT | Chest pain |
| 161972006 | Central chest pain (finding) | SNOMED-CT | Chest pain |
| 161973001 | Anterior chest wall pain (finding) | SNOMED-CT | Chest pain |
| 161974007 | Parasternal pain (finding) | SNOMED-CT | Chest pain |
| 161977000 | Costal margin chest pain (finding) | SNOMED-CT | Chest pain |
| 2237002 | Pleuritic pain (finding) | SNOMED-CT | Chest pain |
| 274664007 | Chest pain on breathing (finding) | SNOMED-CT | Chest pain |
| 274668005 | Non-cardiac chest pain (finding) | SNOMED-CT | Chest pain |
| 279019008 | Central crushing chest pain (finding) | SNOMED-CT | Chest pain |
| 281245003 | Musculoskeletal chest pain (finding) | SNOMED-CT | Chest pain |
| 285385002 | Left sided chest pain (finding) | SNOMED-CT | Chest pain |
| 285386001 | Right sided chest pain (finding) | SNOMED-CT | Chest pain |
| 285389008 | Upper chest pain (finding) | SNOMED-CT | Chest pain |
| 29857009 | Chest pain (finding) | SNOMED-CT | Chest pain |
| 298731003 | Pain of sternum (finding) | SNOMED-CT | Chest pain |
| 3368006 | Dull chest pain (finding) | SNOMED-CT | Chest pain |
| 36859004 | Esophageal chest pain (finding) | SNOMED-CT | Chest pain |
| 371030007 | Squeezing chest pain (finding) | SNOMED-CT | Chest pain |
| 4568003 | Retrosternal pain (finding) | SNOMED-CT | Chest pain |
| 59139008 | Crushing chest pain (finding) | SNOMED-CT | Chest pain |
| 71884009 | Precordial pain (finding) | SNOMED-CT | Chest pain |
| 7921000119104 | Anterior pleuritic pain (finding) | SNOMED-CT | Chest pain |
| 81953000 | Chest pain on exertion (finding) | SNOMED-CT | Chest pain |
| 9267009 | Chest pain at rest (finding) | SNOMED-CT | Chest pain |
| 95421005 | Intercostal myalgia (finding) | SNOMED-CT | Chest pain |
| 97001000119106 | Localized chest pain (finding) | SNOMED-CT | Chest pain |
| R05.1 | Acute cough | ICD-10-CM | Cough |
| R05.2 | Subacute cough | ICD-10-CM | Cough |
| R05.3 | Chronic cough | ICD-10-CM | Cough |
| R05.4 | Cough syncope | ICD-10-CM | Cough |
| R05.8 | Other specified cough | ICD-10-CM | Cough |
| R05.9 | Cough, unspecified | ICD-10-CM | Cough |
| 786.2 | Cough | ICD-9-CM | Cough |
| 786.4 | Abnormal sputum | ICD-9-CM | Cough |
| 111962006 | Postural cough (finding) | SNOMED-CT | Cough |
| 11833005 | Dry cough (finding) | SNOMED-CT | Cough |
| 1204369007 | Unexplained chronic cough (disorder) | SNOMED-CT | Cough |
| 135883003 | Cough with fever (finding) | SNOMED-CT | Cough |
| 161923004 | Productive cough -clear sputum (finding) | SNOMED-CT | Cough |
| 161924005 | Productive cough -green sputum (finding) | SNOMED-CT | Cough |
| 161925006 | Productive cough-yellow sputum (finding) | SNOMED-CT | Cough |
| 161927003 | Night cough present (situation) | SNOMED-CT | Cough |
| 161932002 | Morning cough (finding) | SNOMED-CT | Cough |
| 161933007 | Evening cough (finding) | SNOMED-CT | Cough |
| 161947006 | Nocturnal cough and wheeze (finding) | SNOMED-CT | Cough |
| 162480005 | Cough aggravates symptom (finding) | SNOMED-CT | Cough |
| 17986004 | Barking cough (finding) | SNOMED-CT | Cough |
| 19282004 | Cough on exercise (finding) | SNOMED-CT | Cough |
| 20670007 | Brassy cough (finding) | SNOMED-CT | Cough |
| 247410004 | Painful cough (finding) | SNOMED-CT | Cough |
| 248589007 | Clearing throat - hawking (finding) | SNOMED-CT | Cough |
| 248593001 | Cough when swallowing (finding) | SNOMED-CT | Cough |
| 249596001 | Cough impulse in inguinal canal (finding) | SNOMED-CT | Cough |
| 284523002 | Persistent cough (finding) | SNOMED-CT | Cough |
| 28743005 | Productive cough (finding) | SNOMED-CT | Cough |
| 289114008 | Does cough (finding) | SNOMED-CT | Cough |
| 289965001 | Croupy cough (finding) | SNOMED-CT | Cough |
| 301236000 | Effective cough (finding) | SNOMED-CT | Cough |
| 315246003 | Unexplained cough (finding) | SNOMED-CT | Cough |
| 383350451000119103 | Subacute cough (finding) | SNOMED-CT | Cough |
| 417850002 | Respiratory tract congestion and cough (disorder) | SNOMED-CT | Cough |
| 43025008 | Paroxysmal cough (finding) | SNOMED-CT | Cough |
| 445241004 | Persistent cough after viral respiratory infection (disorder) | SNOMED-CT | Cough |
| 46789001 | Cough after eating (finding) | SNOMED-CT | Cough |
| 49727002 | Cough (finding) | SNOMED-CT | Cough |
| 59994004 | Hacking cough (finding) | SNOMED-CT | Cough |
| 62427007 | Bovine cough (finding) | SNOMED-CT | Cough |
| 62548007 | Nocturnal cough (finding) | SNOMED-CT | Cough |
| 62618004 | Early morning cough (finding) | SNOMED-CT | Cough |
| 62731002 | Increasing frequency of cough (finding) | SNOMED-CT | Cough |
| 63000007 | Spasmodic cough (finding) | SNOMED-CT | Cough |
| 68154008 | Chronic cough (finding) | SNOMED-CT | Cough |
| 7142008 | Cough at rest (finding) | SNOMED-CT | Cough |
| 788062371000119104 | Acute cough (finding) | SNOMED-CT | Cough |
| R61 | Generalized hyperhidrosis | ICD-10-CM | Diaphoresis |
| 780.8 | Generalized hyperhidrosis | ICD-9-CM | Diaphoresis |
| 274673004 | Generalized hyperhidrosis (disorder) | SNOMED-CT | Diaphoresis |
| 52613005 | Excessive sweating (finding) | SNOMED-CT | Diaphoresis |
| R42 | Dizziness and giddiness | ICD-10-CM | Dizziness |
| 780.4 | Dizziness and giddiness | ICD-9-CM | Dizziness |
| 103017008 | Postural dizziness (finding) | SNOMED-CT | Dizziness |
| 103018003 | Exertional dizziness (finding) | SNOMED-CT | Dizziness |
| 103293001 | Persistent postural perceptual dizziness (finding) | SNOMED-CT | Dizziness |
| 249988004 | Oscillation of surroundings (finding) | SNOMED-CT | Dizziness |
| 271789005 | Dizziness and giddiness (finding) | SNOMED-CT | Dizziness |
| 315018008 | Dizzy spells (finding) | SNOMED-CT | Dizziness |
| 404640003 | Dizziness (finding) | SNOMED-CT | Dizziness |
| 407645004 | Dizziness on standing up (finding) | SNOMED-CT | Dizziness |
| R06.00 | Dyspnea, unspecified | ICD-10-CM | Dyspnea |
| R06.01 | Orthopnea | ICD-10-CM | Dyspnea |
| R06.02 | Shortness of breath | ICD-10-CM | Dyspnea |
| R06.03 | Acute respiratory distress | ICD-10-CM | Dyspnea |
| R06.09 | Other forms of dyspnea | ICD-10-CM | Dyspnea |
| R06.8 | Other abnormalities of breathing | ICD-10-CM | Dyspnea |
| R06.89 | Other abnormalities of breathing | ICD-10-CM | Dyspnea |
| R06.9 | Unspecified abnormalities of breathing | ICD-10-CM | Dyspnea |
| 786.00 | Respiratory abnormality, unspecified | ICD-9-CM | Dyspnea |
| 786.02 | Orthopnea | ICD-9-CM | Dyspnea |
| 786.05 | Shortness of breath | ICD-9-CM | Dyspnea |
| 786.09 | Other respiratory abnormalities | ICD-9-CM | Dyspnea |
| 786.09 | Other respiratory abnormalities | ICD-9-CM | Dyspnea |
| 786.02 | Orthopnea | ICD-9-CM | Dyspnea |
| 786.05 | Shortness of breath | ICD-9-CM | Dyspnea |
| 786.9 | Other symptoms involving respiratory system and chest | ICD-9-CM | Dyspnea |
| 786.00 | Respiratory abnormality, unspecified | ICD-9-CM | Dyspnea |
| 25209001 | Inspiratory dyspnea (finding) | SNOMED-CT | Dyspnea |
| 267036007 | Dyspnea (finding) | SNOMED-CT | Dyspnea |
| 397702004 | Borg Breathlessness Score finding (finding) | SNOMED-CT | Dyspnea |
| 55442000 | Paroxysmal nocturnal dyspnea (finding) | SNOMED-CT | Dyspnea |
| 60845006 | Dyspnea on exertion (finding) | SNOMED-CT | Dyspnea |
| 719415004 | Breathlessness care (regime/therapy) | SNOMED-CT | Dyspnea |
| 73322006 | Dyspnea, class IV (finding) | SNOMED-CT | Dyspnea |
| R53 | Malaise and fatigue | ICD-10-CM | Fatigue |
| R53.1 | Weakness | ICD-10-CM | Fatigue |
| R53.8 | Other malaise and fatigue | ICD-10-CM | Fatigue |
| R53.81 | Other malaise | ICD-10-CM | Fatigue |
| R53.83 | Other fatigue | ICD-10-CM | Fatigue |
| 780.79 | Other malaise and fatigue | ICD-9-CM | Fatigue |
| 799.3 | Debility, unspecified | ICD-9-CM | Fatigue |
| 1131000175106 | Asthenia due to disease (finding) | SNOMED-CT | Fatigue |
| 13791008 | Asthenia (finding) | SNOMED-CT | Fatigue |
| 162236007 | Weakness present (finding) | SNOMED-CT | Fatigue |
| 248278004 | Attacks of weakness (finding) | SNOMED-CT | Fatigue |
| 271795006 | Malaise and fatigue (finding) | SNOMED-CT | Fatigue |
| 367391008 | Malaise (finding) | SNOMED-CT | Fatigue |
| 84229001 | Fatigue (finding) | SNOMED-CT | Fatigue |
| R00.2 | Palpitations | ICD-10-CM | Palpitations |
| 785.1 | Palpitations | ICD-9-CM | Palpitations |
| R60 | Edema, not elsewhere classified | ICD-10-CM | Peripheral edema |
| R60.0 | Localized edema | ICD-10-CM | Peripheral edema |
| R60.1 | Generalized edema | ICD-10-CM | Peripheral edema |
| R60.9 | Edema, unspecified | ICD-10-CM | Peripheral edema |
| 782.3 | Edema | ICD-9-CM | Peripheral edema |
| 234176004 | Idiopathic fluid retention (finding) | SNOMED-CT | Peripheral edema |
| 248499004 | Dependent edema (finding) | SNOMED-CT | Peripheral edema |
| 267038008 | Edema (finding) | SNOMED-CT | Peripheral edema |
| 284521000 | Pitting edema (finding) | SNOMED-CT | Peripheral edema |
| 420829009 | 1+ pitting edema (finding) | SNOMED-CT | Peripheral edema |
| 421129002 | 4+ pitting edema (finding) | SNOMED-CT | Peripheral edema |
| 421346005 | 3+ pitting edema (finding) | SNOMED-CT | Peripheral edema |
| 421605005 | 2+ pitting edema (finding) | SNOMED-CT | Peripheral edema |
| 43498006 | Body fluid retention (finding) | SNOMED-CT | Peripheral edema |
| 442433009 | Anasarca (finding) | SNOMED-CT | Peripheral edema |
| 445281006 | Soft pitting edema (finding) | SNOMED-CT | Peripheral edema |
| 445341001 | Firm pitting edema (finding) | SNOMED-CT | Peripheral edema |
| 445345005 | Remitting seronegative symmetrical synovitis with pitting edema (disorder) | SNOMED-CT | Peripheral edema |
| 721181006 | Localized edema due to fluid overload (disorder) | SNOMED-CT | Peripheral edema |
| 721210009 | Edema due to fluid overload (disorder) | SNOMED-CT | Peripheral edema |
| 789007001 | Pitting edema of lip (finding) | SNOMED-CT | Peripheral edema |
| 860797003 | Generalized edema due to fluid overload (disorder) | SNOMED-CT | Peripheral edema |

Table S5. Troponin T value set

| **Code** | **Description** | **Vocabulary** |
| --- | --- | --- |
| 48426-1 | Troponin T.cardiac [Presence] in Blood | LOINC |
| 48425-3 | Troponin T.cardiac [Mass/volume] in Blood | LOINC |
| 33204-9 | Troponin T.cardiac [Presence] in Serum or Plasma | LOINC |
| 6597-9 | Troponin T.cardiac [Mass/volume] in Venous blood | LOINC |
| 101915-7 | Troponin T.cardiac [Mass/volume] in 6 hour Serum or Plasma | LOINC |
| 6598-7 | Troponin T.cardiac [Mass/volume] in Serum or Plasma | LOINC |
| 89575-5 | Troponin T.cardiac [Interpretation] in Serum or Plasma Qualitative by High sensitivity method | LOINC |
| 67151-1 | Troponin T.cardiac [Mass/volume] in Serum or Plasma by High sensitivity method | LOINC |
| 89576-3 | Troponin T.cardiac panel - Serum or Plasma by High sensitivity method | LOINC |
| 101865-4 | Troponin T. cardiac 2H and 6H 5th generation panel in Serum or Plasma by High sensitivity method | LOINC |
| 101911-6 | Troponin T. cardiac [Mass/volume] in 2 hour 5th generation Serum or Plasma | LOINC |
| 105000003 | Troponin measurement (procedure) | SNOMED-CT |
| 121871002 | Troponin T measurement (procedure) | SNOMED-CT |
| 314068007 | Plasma troponin T measurement (procedure) | SNOMED-CT |
| 166794009 | Serum troponin T measurement (procedure) | SNOMED-CT |
| 105001004 | Troponin T cardiac measurement (procedure) | SNOMED-CT |
| 144070007 | Serum troponin T level (procedure) | SNOMED-CT |
| 144075002 | Plasma troponin T level (procedure) | SNOMED-CT |
| 166799004 | Plasma troponin T level (procedure) | SNOMED-CT |
| 84512 | Troponin, qualitative | CPT |
| 84484 | Troponin, quantitative | CPT |

Table S6. Troponin I value set

| **Code** | **Description** | **Vocabulary** |
| --- | --- | --- |
| 42757-5 | Troponin I.cardiac [Mass/volume] in Blood | LOINC |
| 16255-2 | Troponin I.cardiac [Units/volume] in Serum or Plasma | LOINC |
| 10839-9 | Troponin I.cardiac [Mass/volume] in Serum or Plasma | LOINC |
| 89578-9 | Troponin I.cardiac [Interpretation] in Serum or Plasma Qualitative by High sensitivity method | LOINC |
| 89579-7 | Troponin I.cardiac [Mass/volume] in Serum or Plasma by High sensitivity method | LOINC |
| 89577-1 | Troponin I.cardiac panel - Serum or Plasma by High sensitivity method | LOINC |
| 49563-0 | Troponin I.cardiac [Mass/volume] in Serum or Plasma by Detection limit <= 0.01 ng/mL | LOINC |
| 76399-5 | Troponin I.cardiac [Presence] in Serum, Plasma or Blood by Rapid immunoassay | LOINC |
| 444931001 | Troponin I above reference range (finding) | SNOMED-CT |
| 105000003 | Troponin measurement (procedure) | SNOMED-CT |
| 121870001 | Troponin I measurement (procedure) | SNOMED-CT |
| 313724009 | Plasma troponin I measurement (procedure) | SNOMED-CT |
| 313616005 | Serum troponin I measurement (procedure) | SNOMED-CT |
| 144074003 | Serum troponin I level (procedure) | SNOMED-CT |
| 166798007 | Serum troponin I level (procedure) | SNOMED-CT |
| 144072004 | Plasma troponin I level (procedure) | SNOMED-CT |
| 166796006 | Plasma troponin I level (procedure) | SNOMED-CT |
| 84512 | Troponin, qualitative | CPT |
| 84484 | Troponin, quantitative | CPT |

Table S7. Creatine kinase myocardial band value set

| **Code** | **Description** | **Vocabulary** |
| --- | --- | --- |
| 104613001 | Creatine kinase MB isoenzyme measurement (procedure) | SNOMED-CT |
| 250736004 | Creatine kinase MB isoenzyme/total creatine kinase ratio measurement (procedure) | SNOMED-CT |
| 408250001 | Plasma creatine kinase MB isoenzyme level (procedure) | SNOMED-CT |
| 166672007 | Serum creatine kinase MB isoenzyme measurement (procedure) | SNOMED-CT |
| 12016004 | Creatine kinase isoenzyme, MB fraction (substance) | SNOMED-CT |
| 144375005 | MB/total creatine kinase ratio (procedure) | SNOMED-CT |
| 167136005 | MB/total creatine kinase ratio (procedure) | SNOMED-CT |
| 82553 | Creatine kinase (CK), (CPK); MB fraction only \| [Laboratory Procedure] | CPT |
| 13969-1 | Creatine kinase.MB [Mass/volume] in Serum or Plasma | LOINC |
| 49551-5 | Creatine kinase.MB [Mass/volume] in Blood | LOINC |
| 83092-7 | Creatine kinase.MB [Mass/volume] in Serum or Plasma by Immunoassay | LOINC |
| 12187-1 | Creatine kinase.MB/Creatine kinase.total in Serum or Plasma by Electrophoresis | LOINC |
| 12188-9 | Deprecated Creatine kinase.MB/Creatine kinase.total | LOINC |
| 20569-0 | Creatine kinase.MB/Creatine kinase.total in Serum or Plasma | LOINC |
| 2158-4 | Creatine kinase.total/Creatine kinase.MB [Enzymatic activity ratio] in Serum or Plasma | LOINC |
| 50757-4 | Creatine kinase.total/Creatine kinase.MB [Enzymatic activity ratio] in Blood | LOINC |
| 2154-3 | Creatine kinase.MB [Enzymatic activity/volume] in Serum or Plasma by Electrophoresis | LOINC |
| 32673-6 | Creatine kinase.MB [Enzymatic activity/volume] in Serum or Plasma | LOINC |
| 6773-6 | Deprecated Creatine kinase.MB | LOINC |
| 101446-3 | Creatine kinase.MB [Moles/volume] in Serum or Plasma by Immunoassay | LOINC |
| 83092-7 | Creatine kinase.MB [Mass/volume] in Serum or Plasma by Immunoassay | LOINC |
| 49136-5 | Creatine kinase.MB/Creatine kinase.total [Ratio] in Serum or Plasma | LOINC |
| 20569-0 | Creatine kinase.MB/Creatine kinase.total in Serum or Plasma | LOINC |
| 12189-7 | Creatine kinase.MB/Creatine kinase.total in Serum or Plasma by calculation | LOINC |
| 72564-8 | Creatine kinase.MB/Creatine kinase.total [Pure catalytic fraction] in Serum or Plasma by calculation | LOINC |
| 72563-0 | Creatine kinase.MB/Creatine kinase.total [Pure catalytic fraction] in Serum or Plasma by Electrophoresis | LOINC |
| 49551-5 | Creatine kinase.MB [Mass/volume] in Blood | LOINC |
| 38482-6 | Creatine kinase.MB [Presence] in Serum or Plasma | LOINC |
| 51506-4 | Creatine kinase.MB [Enzymatic activity/volume] in Cerebral spinal fluid by Electrophoresis | LOINC |

## Myocarditis LLM Prompts

Below are the “prompt templates” used for myocarditis in this project. Prompt templates become actual prompts for specific patients when variables beginning with “$” are swapped out for the actual values for the patient under consideration. In some cases, few shot examples were developed using experience with the train patients and inserted into the prompts (see the line “Examples (if available)” in each prompt, see also Appendices H and I).

### Symptoms and Signs Myocarditis Prompt

Below I will provide you with some patient data.

Using only this data, answer the following question: **Is there evidence that this patient had the abnormal finding of "$finding" some time between $diagnosis_window_start and $diagnosis_window_end: yes or no?**

**Critical Requirements:** - Use ONLY explicitly documented signs/symptoms. Do not infer the presence of findings not directly stated. - Do not use findings from imaging or other diagnostic studies unless explicitly requested. - Do not answer 'yes' if it is merely possible the patient experienced the symptom/sign - there must be explicit documentation. - Do NOT use "indications for a study/lab/procedure" to answer questions about symptoms. For example, if the patient data says "indication for echocardiogram: chest pain, shortness of breath", do NOT consider that as providing reliable evidence of patient symptoms.

**Let's work through this step by step:**

1. **Evidence Identification**: Search the patient data for any mentions of "$finding" or directly related terms 2. **Evidence Evaluation**: For each piece of evidence found: - Quote the relevant text	- Identify the source (note author, date, title/identifier)	- Determine if it explicitly states the finding occurred OR only provides indirect/related information 3. **Temporal Analysis**: If explicit evidence exists, determine if it falls within the specified time window ($diagnosis_window_start to $diagnosis_window_end) 4. **Final Determination**: Based on steps 1-3, provide your yes/no answer

**Examples (if available):** $examples

**IMPORTANT: You must provide your response in the exact JSON format shown below. Do not include any text before or after the JSON. Try to use plain text only in your response, avoid markdown or special characters.**

$response_model

**Patient Data:** $patient_data

### EKG Myocarditis Prompt

Below I will provide you with some patient data.

Using only this data, answer the following question: **Did the patient have an abnormal EKG finding of "$finding" some time between $diagnosis_window_start and $diagnosis_window_end: yes or no?**

**Critical Requirements:** - Use ONLY reported EKG results. Do not infer the presence of findings not explicitly mentioned in EKG reports. - If the finding is not explicitly mentioned in an EKG report, your answer must be "no". - Ignore other information such as patient's final diagnosis and symptoms. - Note: "sinus tachycardia" by itself is not an "arrhythmia", nor is it a "supraventricular tachycardia".

**Let's work through this step by step:**

1. **EKG Report Identification**: Find all EKG reports or results in the patient data 2. **Finding Search**: Search each EKG report for explicit mentions of "$finding" or directly related terms 3. **Evidence Evaluation**: For each relevant EKG finding: - Quote the exact text from the EKG report	- Identify the source (report date, author, identifier)	- Confirm it explicitly states the finding (not just related symptoms or diagnoses) 4. **Temporal Analysis**: If explicit EKG evidence exists, determine if it falls within the specified time window ($diagnosis_window_start to $diagnosis_window_end) 5. **Final Determination**: Based on steps 1-4, provide your yes/no answer

**Examples (if available):** $examples

**IMPORTANT: You must provide your response in the exact JSON format shown below. Do not include any text before or after the JSON. Try to use plain text only in your response, avoid markdown or special characters.**

$response_model

**Patient Data:** $patient_data

### ECHO Myocarditis Prompt

Below I will provide you with some patient data.

Using only this data, answer the following question: **Is there evidence that this patient had the abnormal ECHO cardiogram finding of "$finding" some time between $diagnosis_window_start and $diagnosis_window_end: yes or no?**

**Critical Requirements:** - Use ONLY reported ECHO cardiogram results. Do not infer the presence of findings not explicitly mentioned in ECHO reports. - If the patient ECHO cardiogram data do not specifically mention the finding, then respond 'no'. - Do not use patient symptoms or other patient data to answer the question. - Focus only on specifically reported ECHO cardiogram findings in the patient data.

**Let's work through this step by step:**

1. **ECHO Report Identification**: Find all ECHO cardiogram reports or results in the patient data 2. **Finding Search**: Search each ECHO report for explicit mentions of "$finding" or directly related terms 3. **Evidence Evaluation**: For each relevant ECHO finding: - Quote the exact text from the ECHO report	- Identify the source (report date, author, identifier)	- Confirm it explicitly states the finding (not just related symptoms or diagnoses) 4. **Temporal Analysis**: If explicit ECHO evidence exists, determine if it falls within the specified time window ($diagnosis_window_start to $diagnosis_window_end) 5. **Final Determination**: Based on steps 1-4, provide your yes/no answer

**Examples (if available):** $examples

**IMPORTANT: You must provide your response in the exact JSON format shown below. Do not include any text before or after the JSON. Try to use plain text only in your response, avoid markdown or special characters.**

$response_model

**Patient Data:** $patient_data

### CMRI Myocarditis Prompt

Below I will provide you with some patient data.

Using only this data, answer the following question: **Is there evidence that this patient had the abnormal cardiac MRI finding of "$finding" some time between $diagnosis_window_start and $diagnosis_window_end: yes or no?**

**Critical Requirements:** - Use ONLY reported cardiac MRI results. Do not infer the presence of findings not explicitly mentioned in cardiac MRI reports. - If the patient cardiac MRI data do not specifically mention the finding, then respond 'no'. - Do not use patient symptoms or other patient data to answer the question. - Focus only on specifically reported cardiac MRI findings in the patient data.

**Let's work through this step by step:**

1. **Cardiac MRI Report Identification**: Find all cardiac MRI reports or results in the patient data 2. **Finding Search**: Search each cardiac MRI report for explicit mentions of "$finding" or directly related terms 3. **Evidence Evaluation**: For each relevant cardiac MRI finding: - Quote the exact text from the cardiac MRI report	- Identify the source (report date, author, identifier)	- Confirm it explicitly states the finding (not just related symptoms or diagnoses) 4. **Temporal Analysis**: If explicit cardiac MRI evidence exists, determine if it falls within the specified time window ($diagnosis_window_start to $diagnosis_window_end) 5. **Final Determination**: Based on steps 1-4, provide your yes/no answer

**Examples (if available):** $examples

**IMPORTANT: You must provide your response in the exact JSON format shown below. Do not include any text before or after the JSON. Try to use plain text only in your response, avoid markdown or special characters.**

$response_model

**Patient Data:** $patient_data

### BIOPSY Myocarditis Prompt

Below I will provide you with some patient data.

Using only this data, answer the following question: **Is there evidence that this patient had the abnormal finding of "$finding" some time between $diagnosis_window_start and $diagnosis_window_end: yes or no?**

**Critical Requirements:** - Use ONLY specifically reported histopathologic findings based on histopathologic examination. - If the patient data do not specifically mention the finding based on histopathologic examination, then respond 'no'. - A mere mention of conditions (e.g., 'myocarditis' or 'myocardial inflammation') is not enough to respond 'yes' - the finding must be based specifically on histopathologic examination. - Do not use patient symptoms, discharge diagnoses, or other patient data unrelated to histopathologic examination. - Do not use results from imaging studies such as cardiac MRI or ECHO.

**Let's work through this step by step:**

1. **Histopathologic Report Identification**: Find all histopathologic examination reports, biopsy results, or pathology findings in the patient data 2. **Finding Search**: Search each histopathologic report for explicit mentions of "$finding" or directly related terms 3. **Evidence Evaluation**: For each relevant histopathologic finding: - Quote the exact text from the histopathologic report	- Identify the source (report date, author, identifier)	- Confirm it explicitly states the finding is based on histopathologic examination (not just clinical diagnosis) 4. **Temporal Analysis**: If explicit histopathologic evidence exists, determine if it falls within the specified time window ($diagnosis_window_start to $diagnosis_window_end) 5. **Final Determination**: Based on steps 1-4, provide your yes/no answer

**Examples (if available):** $examples

**IMPORTANT: You must provide your response in the exact JSON format shown below. Do not include any text before or after the JSON. Try to use plain text only in your response, avoid markdown or special characters.**

$response_model

**Patient Data:** $patient_data

### Cardiac Biomarker Myocarditis Prompt

Below I will provide you with some patient data.

Using only this data, answer the following question: **Is there evidence that this patient had an elevated $finding (a value higher than $upper_normal_limit) some time between $diagnosis_window_start and $diagnosis_window_end: yes or no?**

**Critical Requirements:** - You must find an explicit mention of the lab test "$finding" and that its value was greater than $upper_normal_limit to answer 'yes'. - If you find explicit mention of the lab and its value was less than $upper_normal_limit, answer 'no'. - If you cannot find an explicit mention of the lab or no lab value is present, answer 'no'. - Account for the fact that units of the lab reported in the patient data may differ from the threshold given above. - Do not try to infer whether the lab value is elevated based on other lab values or patient data.

**Let's work through this step by step:**

1. **Lab Report Identification**: Find all laboratory results or lab reports in the patient data 2. **Specific Lab Search**: Search for explicit mentions of "$finding" lab test with actual numerical values 3. **Value and Unit Analysis**: For each "$finding" lab result found: - Quote the exact lab result text including value and units	- Identify the source (report date, author, identifier)	- Convert units if necessary to compare with threshold ($upper_normal_limit)	- Determine if the value is greater than, less than, or equal to the threshold 4. **Temporal Analysis**: If elevated lab values exist, determine if they fall within the specified time window ($diagnosis_window_start to $diagnosis_window_end) 5. **Final Determination**: Based on steps 1-4, provide your yes/no answer

**Examples (if available):** $examples

**IMPORTANT: You must provide your response in the exact JSON format shown below. Do not include any text before or after the JSON. Try to use plain text only in your response, avoid markdown or special characters.**

$response_model

**Patient Data:** $patient_data

## Additional Results

Table S8. Confusion matrices for all yes/no questions for the three feature identification methods in test set. NC = not calculated due to simple CQL not tasked with identifying imaging abnormalities and a sudden death symptom.

| Grouping | Gold standard answer | Simple CQL, no | Simple CQL, yes | LLM, no | LLM, yes | Enhanced CQL, no | Enhanced CQL, yes |
| --- | --- | --- | --- | --- | --- | --- | --- |
| All questions | No | 182 | 0 | 372 | 48 | 393 | 27 |
| All questions | Yes | 95 | 23 | 12 | 168 | 17 | 163 |
| Cardiac and non-specific symptoms | No | 135 | 0 | 153 | 7 | 154 | 6 |
| Cardiac and non-specific symptoms | Yes | 89 | 1 | 10 | 80 | 10 | 80 |
| Cardiac biomarkers | No | 47 | 0 | 27 | 20 | 47 | 0 |
| Cardiac biomarkers | Yes | 6 | 22 | 1 | 27 | 6 | 22 |
| ECG abnormalities | No | NC | NC | 46 | 12 | 46 | 12 |
| ECG abnormalities | Yes | NC | NC | 0 | 17 | 0 | 17 |
| ECHO abnormalities | No | NC | NC | 82 | 7 | 82 | 7 |
| ECHO abnormalities | Yes | NC | NC | 1 | 35 | 1 | 35 |
| CMR abnormalities | No | NC | NC | 41 | 1 | 41 | 1 |
| CMR abnormalities | Yes | NC | NC | 0 | 8 | 0 | 8 |
| Cardiac symptom: chest pain | No | 6 | 0 | 6 | 0 | 6 | 0 |
| Cardiac symptom: chest pain | Yes | 18 | 1 | 2 | 17 | 2 | 17 |
| Cardiac symptom: palpitations | No | 20 | 0 | 20 | 0 | 20 | 0 |
| Cardiac symptom: palpitations | Yes | 5 | 0 | 0 | 5 | 0 | 5 |
| Cardiac symptom: dyspnea | No | 10 | 0 | 9 | 1 | 9 | 1 |
| Cardiac symptom: dyspnea | Yes | 15 | 0 | 1 | 14 | 1 | 14 |
| Cardiac symptom: diaphoresis | No | 23 | 0 | 23 | 0 | 23 | 0 |
| Cardiac symptom: diaphoresis | Yes | 2 | 0 | 0 | 2 | 0 | 2 |
| Cardiac symptom: sudden death | No | NC | NC | 24 | 1 | 25 | 0 |
| Cardiac symptom: sudden death | Yes | NC | NC | 0 | 0 | 0 | 0 |
| Non-specific symptom: dizziness or syncope | No | 18 | 0 | 17 | 1 | 17 | 1 |
| Non-specific symptom: dizziness or syncope | Yes | 7 | 0 | 1 | 6 | 1 | 6 |
| Non-specific symptom: abdominal pain | No | 18 | 0 | 15 | 3 | 15 | 3 |
| Non-specific symptom: abdominal pain | Yes | 7 | 0 | 1 | 6 | 1 | 6 |
| Non-specific symptom: fatigue | No | 8 | 0 | 8 | 0 | 8 | 0 |
| Non-specific symptom: fatigue | Yes | 17 | 0 | 2 | 15 | 2 | 15 |
| Non-specific symptom: edema | No | 17 | 0 | 17 | 0 | 17 | 0 |
| Non-specific symptom: edema | Yes | 8 | 0 | 0 | 8 | 0 | 8 |
| Non-specific symptom: cough | No | 15 | 0 | 14 | 1 | 14 | 1 |
| Non-specific symptom: cough | Yes | 10 | 0 | 3 | 7 | 3 | 7 |
| Biomarker: troponin T | No | 19 | 0 | 3 | 16 | 19 | 0 |
| Biomarker: troponin T | Yes | 2 | 4 | 0 | 6 | 2 | 4 |
| Biomarker: troponin I | No | 5 | 0 | 1 | 4 | 5 | 0 |
| Biomarker: troponin I | Yes | 3 | 17 | 1 | 19 | 3 | 17 |
| Biomarker: CK-MB | No | 23 | 0 | 23 | 0 | 23 | 0 |
| Biomarker: CK-MB | Yes | 1 | 1 | 0 | 2 | 1 | 1 |
| ECG: paroxysmal or sustained atrial or ventricular arrhythmias | No | NC | NC | 8 | 6 | 8 | 6 |
| ECG: paroxysmal or sustained atrial or ventricular arrhythmias | Yes | NC | NC | 0 | 11 | 0 | 11 |
| ECG: atrioventricular nodal conduction delays or intraventricular conduction defects | No | NC | NC | 16 | 6 | 16 | 6 |
| ECG: atrioventricular nodal conduction delays or intraventricular conduction defects | Yes | NC | NC | 0 | 3 | 0 | 3 |
| ECG: frequent atrial or ventricular ectopy | No | NC | NC | 22 | 0 | 22 | 0 |
| ECG: frequent atrial or ventricular ectopy | Yes | NC | NC | 0 | 3 | 0 | 3 |
| ECHO: new focal or diffuse left or right ventricular function abnormalities | No | NC | NC | 8 | 2 | 8 | 2 |
| ECHO: new focal or diffuse left or right ventricular function abnormalities | Yes | NC | NC | 0 | 15 | 0 | 15 |
| ECHO: segmental wall motion abnormalities | No | NC | NC | 22 | 0 | 22 | 0 |
| ECHO: segmental wall motion abnormalities | Yes | NC | NC | 1 | 2 | 1 | 2 |
| ECHO: global systolic or diastolic function depression/abnormality | No | NC | NC | 10 | 4 | 10 | 4 |
| ECHO: global systolic or diastolic function depression/abnormality | Yes | NC | NC | 0 | 11 | 0 | 11 |
| ECHO: ventricular dilation | No | NC | NC | 17 | 1 | 17 | 1 |
| ECHO: ventricular dilation | Yes | NC | NC | 0 | 7 | 0 | 7 |
| ECHO: wall thickness change | No | NC | NC | 25 | 0 | 25 | 0 |
| ECHO: wall thickness change | Yes | NC | NC | 0 | 0 | 0 | 0 |
| CMR: edema on T2 weighted study | No | NC | NC | 21 | 1 | 21 | 1 |
| CMR: edema on T2 weighted study | Yes | NC | NC | 0 | 3 | 0 | 3 |
| CMR: late gadolinium enhancement on T1 weighted study | No | NC | NC | 20 | 0 | 20 | 0 |
| CMR: late gadolinium enhancement on T1 weighted study | Yes | NC | NC | 0 | 5 | 0 | 5 |
| Biopsy: myocardial inflammation | No | NC | NC | 23 | 1 | 23 | 1 |
| Biopsy: myocardial inflammation | Yes | NC | NC | 0 | 1 | 0 | 1 |

Table S9. Performance metrics (95% CI) of the three feature identification methods answering the individual yes/no questions related to myocarditis in the test set. NC = not calculated due to 1) simple CQL not tasked with identifying imaging abnormalities or sudden death symptom and 2) no ‘yes’ gold-standard determination for the question.

| Grouping | n | Simple CQL MCC | Simple CQL F1-score | LLM MCC | LLM F1-score | Enhanced CQL MCC | Enhanced CQL F1-score |
| --- | --- | --- | --- | --- | --- | --- | --- |
| Cardiac symptom: chest pain | 25 | 0.11 (0.00 - 0.23) | 0.10 (0.00 - 0.30) | 0.82 (0.56 - 1.00) | 0.94 (0.85 - 1.00) | 0.82 (0.56 - 1.00) | 0.94 (0.85 - 1.00) |
| Cardiac symptom: palpitations | 25 | 0.00 (0.00 - 0.00) | 0.00 (0.00 - 0.00) | 1.00 (1.00 - 1.00) | 1.00 (1.00 - 1.00) | 1.00 (1.00 - 1.00) | 1.00 (1.00 - 1.00) |
| Cardiac symptom: dyspnea | 25 | 0.00 (0.00 - 0.00) | 0.00 (0.00 - 0.00) | 0.83 (0.58 - 1.00) | 0.93 (0.82 - 1.00) | 0.83 (0.58 - 1.00) | 0.93 (0.82 - 1.00) |
| Cardiac symptom: diaphoresis | 25 | 0.00 (0.00 - 0.00) | 0.00 (0.00 - 0.00) | 1.00 (0.00 - 1.00) | 1.00 (0.00 - 1.00) | 1.00 (0.00 - 1.00) | 1.00 (0.00 - 1.00) |
| Cardiac symptom: sudden death | 25 | NC | NC | NC | NC | NC | NC |
| Non-specific symptom: dizziness or syncope | 25 | 0.00 (0.00 - 0.00) | 0.00 (0.00 - 0.00) | 0.80 (0.48 - 1.00) | 0.86 (0.57 - 1.00) | 0.80 (0.48 - 1.00) | 0.86 (0.57 - 1.00) |
| Non-specific symptom: abdominal pain | 25 | 0.00 (0.00 - 0.00) | 0.00 (0.00 - 0.00) | 0.65 (0.28 - 0.92) | 0.75 (0.44 - 0.95) | 0.65 (0.28 - 0.92) | 0.75 (0.44 - 0.95) |
| Non-specific symptom: fatigue | 25 | 0.00 (0.00 - 0.00) | 0.00 (0.00 - 0.00) | 0.84 (0.61 - 1.00) | 0.94 (0.83 - 1.00) | 0.84 (0.61 - 1.00) | 0.94 (0.83 - 1.00) |
| Non-specific symptom: edema | 25 | 0.00 (0.00 - 0.00) | 0.00 (0.00 - 0.00) | 1.00 (1.00 - 1.00) | 1.00 (1.00 - 1.00) | 1.00 (1.00 - 1.00) | 1.00 (1.00 - 1.00) |
| Non-specific symptom: cough | 25 | 0.00 (0.00 - 0.00) | 0.00 (0.00 - 0.00) | 0.67 (0.33 - 0.92) | 0.78 (0.50 - 0.96) | 0.67 (0.33 - 0.92) | 0.78 (0.50 - 0.96) |
| Biomarker: troponin T | 25 | 0.78 (0.43 - 1.00) | 0.80 (0.40 - 1.00) | 0.21 (0.00 - 0.36) | 0.43 (0.17 - 0.63) | 0.78 (0.43 - 1.00) | 0.80 (0.40 - 1.00) |
| Biomarker: troponin I | 25 | 0.73 (0.45 - 1.00) | 0.92 (0.81 - 1.00) | 0.22 (-0.16 - 0.69) | 0.88 (0.77 - 0.98) | 0.73 (0.45 - 1.00) | 0.92 (0.81 - 1.00) |
| Biomarker: CK-MB | 25 | 0.69 (0.00 - 1.00) | 0.67 (0.00 - 1.00) | 1.00 (0.00 - 1.00) | 1.00 (0.00 - 1.00) | 0.69 (0.00 - 1.00) | 0.67 (0.00 - 1.00) |
| ECG: paroxysmal or sustained atrial or ventricular arrhythmias | 25 | NC | NC | 0.61 (0.39 - 0.85) | 0.79 (0.58 - 0.93) | 0.61 (0.39 - 0.85) | 0.79 (0.58 - 0.93) |
| ECG: atrioventricular nodal conduction delays or intraventricular conduction defects | 25 | NC | NC | 0.49 (0.00 - 0.76) | 0.50 (0.00 - 0.80) | 0.49 (0.00 - 0.76) | 0.50 (0.00 - 0.80) |
| ECG: frequent atrial or ventricular ectopy | 25 | NC | NC | 1.00 (0.00 - 1.00) | 1.00 (0.00 - 1.00) | 1.00 (0.00 - 1.00) | 1.00 (0.00 - 1.00) |
| ECHO: new focal or diffuse left or right ventricular function abnormalities | 25 | NC | NC | 0.84 (0.61 - 1.00) | 0.94 (0.83 - 1.00) | 0.84 (0.61 - 1.00) | 0.94 (0.83 - 1.00) |
| ECHO: segmental wall motion abnormalities | 25 | NC | NC | 0.80 (0.00 - 1.00) | 0.80 (0.00 - 1.00) | 0.80 (0.00 - 1.00) | 0.80 (0.00 - 1.00) |
| ECHO: global systolic or diastolic function depression/abnormality | 25 | NC | NC | 0.72 (0.51 - 0.92) | 0.85 (0.67 - 0.97) | 0.72 (0.51 - 0.92) | 0.85 (0.67 - 0.97) |
| ECHO: ventricular dilation | 25 | NC | NC | 0.91 (0.70 - 1.00) | 0.93 (0.73 - 1.00) | 0.91 (0.70 - 1.00) | 0.93 (0.73 - 1.00) |
| ECHO: wall thickness change | 25 | NC | NC | NC | NC | NC | NC |
| CMR: edema on T2 weighted study | 25 | NC | NC | 0.85 (0.00 - 1.00) | 0.86 (0.00 - 1.00) | 0.85 (0.00 - 1.00) | 0.86 (0.00 - 1.00) |
| CMR: late gadolinium enhancement on T1 weighted study | 25 | NC | NC | 1.00 (1.00 - 1.00) | 1.00 (1.00 - 1.00) | 1.00 (1.00 - 1.00) | 1.00 (1.00 - 1.00) |
| Biopsy: myocardial inflammation | 25 | NC | NC | 0.69 (0.00 - 1.00) | 0.67 (0.00 - 1.00) | 0.69 (0.00 - 1.00) | 0.67 (0.00 - 1.00) |

## Sensitivity Analyses

Table S10. Sensitivity analysis 1: Performance metrics (95% CI) of the three feature identification methods answering yes/no questions related to myocarditis in the test set, not correcting the gold-standard.

| **Method** | **Set (number of questions)** | **MCC (95% CI)** | **F1-score (95% CI)** |
| --- | --- | --- | --- |
| **Simple CQL** | Optimization (n=120) | 0.32 (0.04 - 0.55) | 0.32 (0.10 - 0.54) |
| **Simple CQL** | Test (n=300) | 0.34 (0.28 - 0.40) | 0.32 (0.27 - 0.38) |
| **LLM** | Optimization (n=240) | 0.47 (0.38 - 0.56) | 0.54 (0.41 - 0.61) |
| **LLM** | Test (n=600) | 0.70 (0.64 - 0.75) | 0.78 (0.73 - 0.83) |
| **Enhanced CQL** | Optimization (n=240) | 0.48 (0.35 - 0.61) | 0.55 (0.41 - 0.66) |
| **Enhanced CQL** | Test (n=600) | 0.74 (0.67 - 0.80) | 0.81 (0.76 - 0.86) |

Table S11. Sensitivity Analysis 1: Performance metrics (95% CI) of the three feature identification methods answering yes/no questions grouped by clinical concepts related to myocarditis in the test set. NC = not calculated due to simple CQL not tasked with identifying imaging abnormalities.

| Grouping | Simple CQL | | LLM | | Enhanced CQL | |
| --- | --- | --- | --- | --- | --- | --- |
|  | MCC (95% CI) | F1-score (95% CI) | MCC (95% CI) | F1-score (95% CI) | MCC (95% CI) | F1-score (95% CI) |
| Cardiac and non-specific symptoms | 0.08 (0.00 - 0.15) | 0.02 (0.00 - 0.07) | 0.82 (0.72 - 0.89) | 0.88 (0.81 - 0.93) | 0.82 (0.74 - 0.89) | 0.89 (0.82 - 0.93) |
| Cardiac biomarkers | 0.80 (0.67 - 0.91) | 0.86 (0.75 - 0.94) | 0.52 (0.44 - 0.61) | 0.70 (0.65 - 0.76) | 0.80 (0.67 - 0.91) | 0.86 (0.75 - 0.94) |
| ECG abnormalities | NC | NC | 0.36 (0.14 - 0.55) | 0.48 (0.28 - 0.64) | 0.36 (0.14 - 0.55) | 0.48 (0.28 - 0.64) |
| ECHO abnormalities | NC | NC | 0.77 (0.64 - 0.88) | 0.83 (0.71 - 0.91) | 0.77 (0.64 - 0.88) | 0.83 (0.71 - 0.91) |
| CMR abnormalities | NC | NC | 0.79 (0.58 - 1.00) | 0.80 (0.55 - 1.00) | 0.79 (0.58 - 1.00) | 0.80 (0.55 - 1.00) |

Table S12. Sensitivity Analysis 1: Performance metrics (95% CI) of the three feature identification methods answering the individual yes/no questions related to myocarditis in the test set. NC = not calculated due to 1) simple CQL not tasked with identifying imaging abnormalities or sudden death symptom and 2) no ‘yes’ gold-standard determination for the question.

| Grouping | n | Simple CQL MCC | Simple CQL F1-score | LLM MCC | LLM F1-score | Enhanced CQL MCC | Enhanced CQL F1-score |
| --- | --- | --- | --- | --- | --- | --- | --- |
| Cardiac symptom: chest pain | 25 | 0.13 (0.00 - 0.25) | 0.11 (0.00 - 0.32) | 0.72 (0.36 - 1.00) | 0.91 (0.80 - 1.00) | 0.72 (0.36 - 1.00) | 0.91 (0.80 - 1.00) |
| Cardiac symptom: palpitations | 25 | 0.00 (0.00 - 0.00) | 0.00 (0.00 - 0.00) | 0.89 (0.63 - 1.00) | 0.91 (0.60 - 1.00) | 0.89 (0.63 - 1.00) | 0.91 (0.60 - 1.00) |
| Cardiac symptom: dyspnea | 25 | 0.00 (0.00 - 0.00) | 0.00 (0.00 - 0.00) | 0.83 (0.58 - 1.00) | 0.93 (0.82 - 1.00) | 0.83 (0.58 - 1.00) | 0.93 (0.82 - 1.00) |
| Cardiac symptom: diaphoresis | 25 | 0.00 (0.00 - 0.00) | 0.00 (0.00 - 0.00) | 0.69 (0.00 - 1.00) | 0.67 (0.00 - 1.00) | 0.69 (0.00 - 1.00) | 0.67 (0.00 - 1.00) |
| Cardiac symptom: sudden death | 25 | NC | NC | NC | NC | NC | NC |
| Non-specific symptom: dizziness or syncope | 25 | 0.00 (0.00 - 0.00) | 0.00 (0.00 - 0.00) | 0.80 (0.48 - 1.00) | 0.86 (0.57 - 1.00) | 0.80 (0.48 - 1.00) | 0.86 (0.57 - 1.00) |
| Non-specific symptom: abdominal pain | 25 | 0.00 (0.00 - 0.00) | 0.00 (0.00 - 0.00) | 0.65 (0.28 - 0.92) | 0.75 (0.44 - 0.95) | 0.65 (0.28 - 0.92) | 0.75 (0.44 - 0.95) |
| Non-specific symptom: fatigue | 25 | 0.00 (0.00 - 0.00) | 0.00 (0.00 - 0.00) | 0.75 (0.43 - 1.00) | 0.90 (0.77 - 1.00) | 0.75 (0.43 - 1.00) | 0.90 (0.77 - 1.00) |
| Non-specific symptom: edema | 25 | 0.00 (0.00 - 0.00) | 0.00 (0.00 - 0.00) | 1.00 (1.00 - 1.00) | 1.00 (1.00 - 1.00) | 1.00 (1.00 - 1.00) | 1.00 (1.00 - 1.00) |
| Non-specific symptom: cough | 25 | 0.00 (0.00 - 0.00) | 0.00 (0.00 - 0.00) | 0.67 (0.33 - 0.92) | 0.78 (0.50 - 0.96) | 0.67 (0.33 - 0.92) | 0.78 (0.50 - 0.96) |
| Biomarker: troponin T | 25 | 0.78 (0.43 - 1.00) | 0.80 (0.40 - 1.00) | 0.21 (0.00 - 0.36) | 0.43 (0.17 - 0.63) | 0.78 (0.43 - 1.00) | 0.80 (0.40 - 1.00) |
| Biomarker: troponin I | 25 | 0.73 (0.45 - 1.00) | 0.92 (0.81 - 1.00) | 0.22 (-0.16 - 0.69) | 0.88 (0.77 - 0.98) | 0.73 (0.45 - 1.00) | 0.92 (0.81 - 1.00) |
| Biomarker: CK-MB | 25 | -0.04 (-0.11 - 0.00) | 0.00 (0.00 - 0.00) | 0.69 (0.00 - 1.00) | 0.67 (0.00 - 1.00) | -0.04 (-0.11 - 0.00) | 0.00 (0.00 - 0.00) |
| ECG: paroxysmal or sustained atrial or ventricular arrhythmias | 25 | NC | NC | 0.09 (-0.32 - 0.46) | 0.57 (0.32 - 0.76) | 0.09 (-0.32 - 0.46) | 0.57 (0.32 - 0.76) |
| ECG: atrioventricular nodal conduction delays or intraventricular conduction defects | 25 | NC | NC | 0.39 (0.00 - 0.66) | 0.36 (0.00 - 0.67) | 0.39 (0.00 - 0.66) | 0.36 (0.00 - 0.67) |
| ECG: frequent atrial or ventricular ectopy | 25 | NC | NC | NC | NC | NC | NC |
| ECHO: new focal or diffuse left or right ventricular function abnormalities | 25 | NC | NC | 0.77 (0.54 - 1.00) | 0.90 (0.77 - 1.00) | 0.77 (0.54 - 1.00) | 0.90 (0.77 - 1.00) |
| ECHO: segmental wall motion abnormalities | 25 | NC | NC | 0.80 (0.00 - 1.00) | 0.80 (0.00 - 1.00) | 0.80 (0.00 - 1.00) | 0.80 (0.00 - 1.00) |
| ECHO: global systolic or diastolic function depression/abnormality | 25 | NC | NC | 0.67 (0.45 - 0.85) | 0.80 (0.59 - 0.95) | 0.67 (0.45 - 0.85) | 0.80 (0.59 - 0.95) |
| ECHO: ventricular dilation | 25 | NC | NC | 0.73 (0.44 - 1.00) | 0.77 (0.40 - 1.00) | 0.73 (0.44 - 1.00) | 0.77 (0.40 - 1.00) |
| ECHO: wall thickness change | 25 | NC | NC | 0.00 (0.00 - 0.00) | 0.00 (0.00 - 0.00) | 0.00 (0.00 - 0.00) | 0.00 (0.00 - 0.00) |
| CMR: edema on T2 weighted study | 25 | NC | NC | 0.47 (0.00 - 0.89) | 0.40 (0.00 - 1.00) | 0.47 (0.00 - 0.89) | 0.40 (0.00 - 1.00) |
| CMR: late gadolinium enhancement on T1 weighted study | 25 | NC | NC | 1.00 (1.00 - 1.00) | 1.00 (1.00 - 1.00) | 1.00 (1.00 - 1.00) | 1.00 (1.00 - 1.00) |
| Biopsy: myocardial inflammation | 25 | NC | NC | 0.46 (-0.09 - 1.00) | 0.50 (0.00 - 1.00) | 0.46 (-0.09 - 1.00) | 0.50 (0.00 - 1.00) |

Table S13. Sensitivity Analysis 1: Confusion matrices comparing LLM, enhanced CQL, and gold-standard outcome determinations in the test set

| Method | Outcome determination | Gold-standard | | |
| --- | --- | --- | --- | --- |
|  |  | **No** | **Probable** | **Definite** |
| **LLM** | **No** | 0 | 1 | 0 |
|  | **Probable** | 0 | 7 | 0 |
|  | **Definite** | 1 | 2 | 14 |
| **Enhanced CQL** | **No** | 0 | 2 | 0 |
|  | **Probable** | 1 | 6 | 0 |
|  | **Definite** | 0 | 2 | 14 |

Table S14. Sensitivity analysis 2: performance metrics (95% CI) of the LLM and enhanced CQL feature extraction methods answering all yes/no questions related to myocarditis except for the ECG abnormality questions in the test set.

| **Method** | **Set (number of questions)** | **MCC (95% CI)** | **F1-score (95% CI)** |
| --- | --- | --- | --- |
| **LLM** | Optimization (n=210) | 0.64 (0.54 - 0.72) | 0.67 (0.54 - 0.75) |
| **LLM** | Test (n=525) | 0.80 (0.74 - 0.85) | 0.86 (0.82 - 0.90) |
| **Enhanced CQL** | Optimization (n=210) | 0.65 (0.50 - 0.79) | 0.69 (0.54 - 0.82) |
| **Enhanced CQL** | Test (n=525) | 0.86 (0.80 - 0.91) | 0.90 (0.86 - 0.94) |

**NOTICE**

This software/technical data was produced for the U. S. Government under Contract Number 75FCMC23D0004, and is subject to Federal Acquisition Regulation Clause 52.227-14, Rights in Data-General.

No other use other than that granted to the U. S. Government, or to those acting on behalf of the U. S. Government under that Clause is authorized without the express written permission of The MITRE Corporation.

For further information, please contact The MITRE Corporation, Contracts Management Office, 7515 Colshire Drive, McLean, VA 22102-7539, (703) 983-6000.

**© 2026 The MITRE Corporation.**
